# A dual-functional flexible sensor based on defects-free Co-doped ZnO nanorods decorated with CoO clusters towards pH and glucose monitoring of fruit juices and human fluids

**DOI:** 10.1186/s40580-022-00305-x

**Published:** 2022-03-22

**Authors:** Muhammad Hilal, Woochul Yang

**Affiliations:** Department of Physics, Dongguk University, Seoul, 04620 Republic of Korea

**Keywords:** CoO/Co-doped ZnO heterostructure, Defect states, Flat band potential, Flexible pH and glucose sensor, Human fluids, Fruit juices

## Abstract

Herein, ZnO nanorods were doped with Co and decorated with CoO clusters through an in situ technique to create a CoO/Co-doped ZnO (CO/CZO) heterostructure at low temperatures (150 °C) on a flexible PET substrate. In the CO/CZO heterostructure, the Co dopant has a low energy barrier to substitute Zn atoms and adsorb over oxygen atoms and their vacancies. Therefore, it decreased the charge density (N_D_ = 2.64 × 10^19^ cm^−3^) on non-active sites of ZnO and lowered the charge transfer resistance (317 Ω) at Co-doped-ZnO/electrolyte interface by suppressing the native defects and reducing the Schottky barrier height (− 0.35 eV), respectively. Furthermore, CoO clusters induced a p-n heterostructure with Co-doped ZnO, prevented corrosion, increased the active sites for analyte absorption, and increased the ultimate tensile strength (4.85 N m^−2^). These characteristics enabled the CO/CZO heterostructure to work as a highly sensitive, chemically stable, and flexible pH and glucose oxidation electrode. Therefore, CO/CZO heterostructure was explored for pH monitoring in human fluids and fruit juices, demonstrating a near-Nernst-limit pH sensitivity (52 mV/pH) and fast response time (19 s) in each human fluid and fruit juice. Also, it demonstrated high sensitivity (4656 µM mM^−1^ cm^−2^), low limit of detection (0.15 µM), a broad linear range (0.04 mM to 8.85 mM) and good anti-interference capacity towards glucose-sensing. Moreover, it demonstrated excellent flexibility performances, retained 53% and 69% sensitivity of the initial value for pH and glucose sensors, respectively, after 500 bending, stretching, and warping cycles.

**Graphical Abstract:**

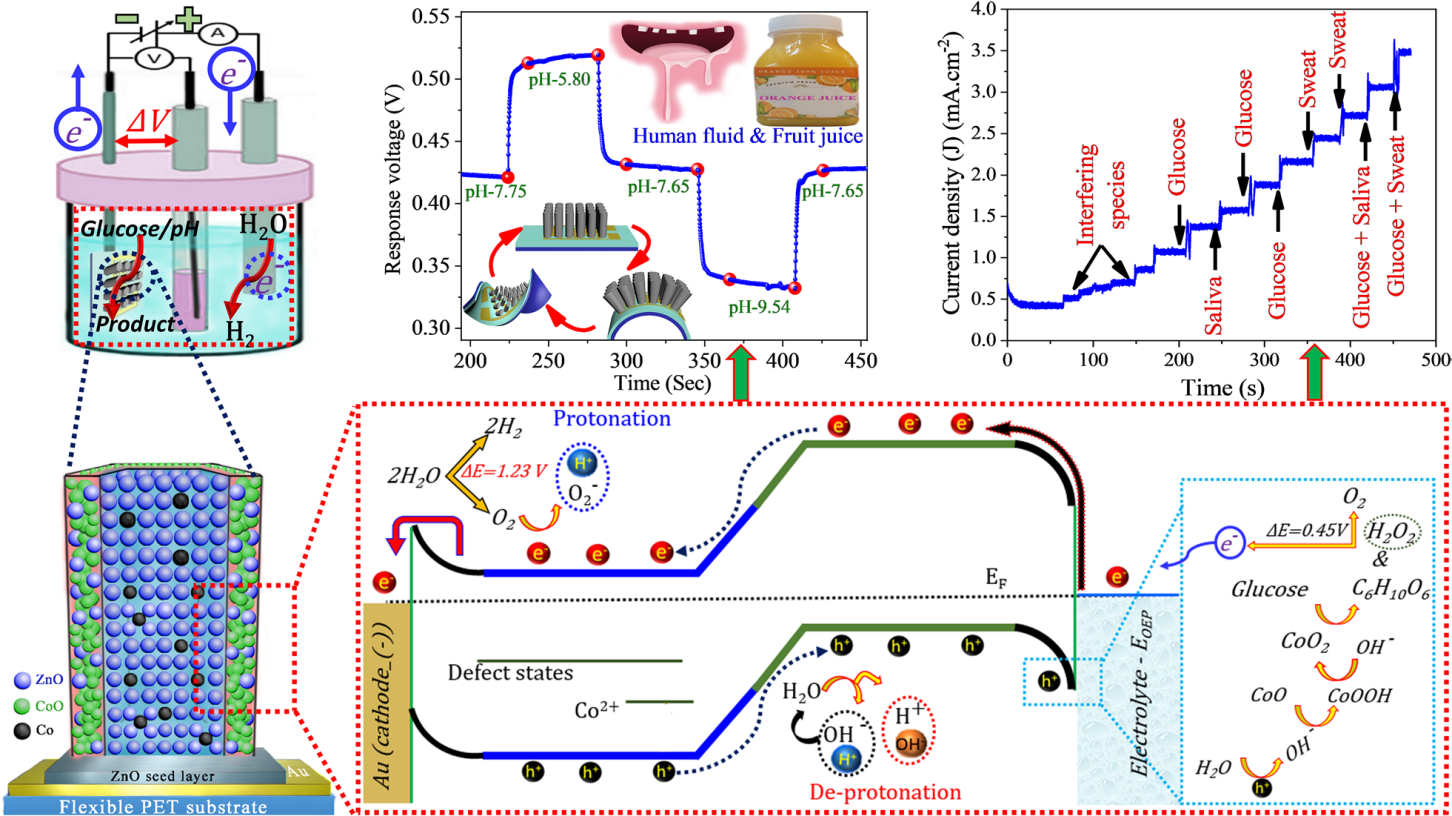

**Supplementary Information:**

The online version contains supplementary material available at 10.1186/s40580-022-00305-x.

## Highlights

- Low-temperatures growth of defect-free CoO/Co-doped ZnO heterostructure.
- Doping lowered N_D_ on non-active sites, ɸ_B_, and R_ct_ at ZnO/electrolyte interface.
- CoO prevent corrosion, increase active sites and enhance ultimate tensile strength.
- Obtained high sensitivity 52 mV/pH for pH and 4656 µM mM^−1^ cm^−2^ for glucose sensors
- Both potentiometric and amperometric mechanism of the heterostructure is proposed.

**Supplementary Information:**

The online version contains supplementary material available at 10.1186/s40580-022-00305-x.

## Introduction

pH and glucose sensors have practical applications in various areas such as research, medicine, wastewater treatment, and agriculture [1–6]. Most commercially available pH sensors are expensive and bulky; therefore, they are not suitable for applications requiring miniature pH sensors [7, 8]. On the other hand, commercial glucose sensors rely on enzymes, which are affected by environmental factors, such as pH of the electrolyte, temperature, and humidity [9, 10]. Thus, the demand for inexpensive, portable, precise commercial pH and non-enzymatic glucose sensors has continued to increase. This demand can be met by metal oxide semiconductors, which offer advantages such as low cost, excellent biocompatibility, and easy integration with wearable devices used in healthcare [11–14]. Several metal-oxide films are currently used as multifunctional sensing applications, such as Rh_2_O_3_ [15] SnO_2_ [16], WO_3_ [17], IGZO [18, 19], ITO [20], Ta_2_O_5_ [21], MgO [22], Al_2_O_3_/SiO_2_ [23], TiO_2_ [24], CuO [13], ZnO [3, 25–29] and CoO [30, 31]. Among these, ZnO has several unique features such as a wide band gap, low intrinsic carrier concentration, high maximum n-type doping, and high maximum p-type doping of 3.32 eV, < 10^6^ cm^−3^, 10^19^ cm^−3^, and 10^–17^ cm^−3^, respectively [32, 33]. ZnO also has the advantages of being non-toxic, biosafe, and biocompatible, and it has high electron communication properties as well as high chemical stability [34, 35]. Additionally, ZnO is an amphoteric material that interacts with both H^+^ and OH^−^ ions in solution, forming surface bonds [36, 37]. The rate of this surface bond formation is proportional to the concentration of free electrons or holes on the active site of ZnO. ZnO with a nanorod-like morphology exhibits a large surface-area-to-volume ratio, which increases its interaction with the target analyte and thereby improves the response time and sensitivity of the corresponding sensor. All these characteristics make ZnO nanorods a suitable material for the development of miniature pH and non-enzymatic glucose sensors. In addition to these characteristics, the band-edge potential of ZnO is also important to determine its performance as an electrochemical sensor. ZnO exhibit more negative and more positive band edge potential of conduction band minima (E_CB_) and valence band maxima (E_VB_) leads to better reduction and oxidation of the reactants in the aqueous electrolyte, respectively. However, the energy levels of native defects, such as oxygen vacancies (O_v_), zinc vacancies (Zn_v_), zinc/oxygen interstitials (Zn/O_in_), and free –OH groups, are positioned within the band gap of ZnO, possessing a lower potential in the range of 0.05–1.8 eV; also called non-active sites [38]. During applied voltage, these defect levels trap charge carriers and hinder their transport toward active sites, preventing them from participating in aqueous electrolyte-based electrochemical reactions and ultimately limiting the use of ZnO in applications such as pH, glucose, caffeine, urea, and photo sensors. Therefore, minimizing the number of defects in ZnO is crucial to meet the requirements of different applications.

ZnO is generally doped with metal ions such as Ti, Ga, Co, Al, Cu, Cr, Fe, Ni, Er, Mg, and Mn to adjust its conductivity to suit the target application [3, 39–49]. For example, several groups have mitigated the defects in ZnO through Mg, Ti, Ga, and Cr doping and improved the sensing performance of ZnO-based pH-, glucose-, urea-, and photo-sensors [39, 44, 46, 50, 51]. Among all the previously explored dopant atoms for ZnO, Co offers many practical advantages. The stability of Co in the divalent oxidation state allows the use of flexible synthesis conditions. Furthermore, because the ionic radii of Co (0.72 Å) and Zn (0.74 Å) are similar, small distortions are produced in the lattice of Co-doped ZnO. Moreover, Co has a low energy barrier to substitute Zn atoms and adsorb over oxygen atoms and their vacancies in ZnO [52]. These useful characteristics of Co as a dopant enhance the charge-carrier interactions with the target analyte in an electrolyte and improve the mechanical property by increasing the charge carrier density on the active site via reduction of defects in ZnO and repairing the lattice structure of ZnO, respectively, thereby enhancing the performance of ZnO based electrochemical sensor. However, Co doping generates additional Co-3d states within the band gap of ZnO [53] at less positive potential (1.19 eV) than the reduction potential, which decreases the band gap of ZnO, facilitates the recombination of free charge carriers, and hinders the transport of charge carriers toward active sites. Therefore, it seems that the contribution of Co as a dopant is not sufficient to significantly improve the sensing properties of ZnO. To overcome these challenges, heterojunction formation of Co-doped ZnO with a p-type semiconductor, i.e., cobalt oxide (CoO), is a suitable approach that induces an internal electric field ($\vec{E}$), which provides the necessary driving force for steering rapidly the free charges in the E_VB_ of CoO from the E_VB_ of ZnO, minimizing the trapping probability of charge carriers in the Co-3d state.

A simple, low-cost, and one-step technique to form the heterostructure is to anneal the Co-doped ZnO to transfer some Co-doped atoms to CoO clusters. As Kyle et al., proved based on density functional theory, that CoO clusters formation is the more energetically favorable configuration in Co-doped ZnO than the random distribution of the Co dopant. Owing to the low binding energy of CoO, i.e., − 1.51 eV over the (1010) surface and − 4.52 eV over the (1120) surface, it has the tendency to segregate and form CoO clusters [54]. However, there is disagreement regarding the favorable conditions for the formation of the Co or CoO phase in ZnO. Previously, researchers incorporated Co in ZnO at higher doping concentrations (> 10 wt.%) and temperatures (≥ 300 °C) but found neither Co nor CoO phases in Co-doped ZnO nanostructures [47, 48, 55–61]. These studies concluded that Co cannot form a second phase with ZnO, because Co is fully incorporated into the host lattice, keeping the wurtzite structure of ZnO unaltered. Furthermore, a few studies have reported that Co can form Co(OH)_2_, CoO, and Co_2_O_3_ phases in ZnO in the temperature range of 100–225 °C [62–64].

The above discussion suggests that most of the challenges in ZnO have been resolved by Co doping. However, the limitations of Co doping still exist, negatively impacting the sensing properties of ZnO based electrochemical sensors. Therefore, it is crucial to minimize the challenges posed by Co doping using a simple and low-cost technique. Additionally, a consensus is yet to be reached on the favorable conditions for the formation of the Co or CoO phase in ZnO for the integration of metal-oxide semiconductors with low-melting-point polymeric substrate–based wearable/flexible devices for use in healthcare. In the present study, ZnO nanorods were doped with Co and decorated with CoO clusters to create a CoO/Co-doped ZnO (CO/CZO) heterostructure at low temperatures (150 °C) for integration with a flexible substrate. The formation of CoO clusters was confirmed by high-resolution transmission electron microscopy (HR-TEM), scanning transmission electron microscopy (STEM), X-ray diffraction (XRD), Raman spectroscopy, UV–visible spectroscopy, absorption spectroscopy, and X-ray photoelectron spectroscopy (XPS). In the CO/CZO heterostructure, Co dopant decreased the charge density to N_D_ = 2.64 × 10^19^ cm^−3^ from 7.16 × 10^19^ cm^−3^ on defect sites and improved the mechanical properties of ZnO by reducing the native defects in ZnO and repairing the defective and relaxed lattice structure of ZnO, respectively. Also, Co dopant lowering the charge transfer resistance from the electrolyte to the ZnO by reducing the Schotty barrier height from − 0.43 eV to − 0.35 eV. Furthermore, CoO clusters minimizing the trapping probability of charge carriers in the Co-3d state, enhancing the number of active sites for post-synthetic reactions. Additionally, CoO clusters prevented corrosion and strengthened the ultimate tensile stress of the Co-doped ZnO nanorods. Owing to these advantages, CO/CZO heterostructures were explored as highly flexible and chemically stable pH electrodes for pH monitoring in human fluids and fruit juices. Also, it demonstrated high sensitivity (4,656 µM mM^−1^ cm^−2^), low limit of detection (0.15 µM), a broad linear range (0.04 mM to 8.85 mM) and good selectivity against various interferents towards glucose-sensing.

## Experimental details

### Low-temperature growth of CoO/ZnO nanorod heterojunction and fabrication of bi-functional sensors

**Fig. 1:**
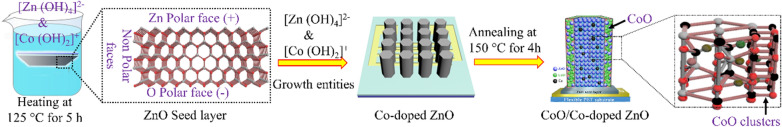
Schematic illustration of the CO/CZO heterostructure nanorods at low temperature on PET substrates

Initially, a patterned interdigitated gold electrode (IDGE) was transferred to a clean and transparent polyethylene terephthalate (PET) substrate. Subsequently, Au was deposited for 60 s using a thermal evaporator to obtain contact electrodes for the constructed pH and glucose sensors. To prepare bare ZnO nanorod–based electrochemical sensors, a ZnO seed layer coating was prepared over the patterned IDGE as follows. An aqueous sol–gel solution was prepared by dissolving Zn(CH_3_COO)_2_·2H_2_O in 1-propanol to obtain a 15 mM solution. The mixture was sonicated for 3 h and then allowed to stabilize at room temperature in an isolated environment. Subsequently, the edge of the Au pattern was masked to spin coat the ZnO seed layer on the center of the pattern at a speed of 2000 rpm for 40 s. The seed layer was then baked for 5 min at 80 °C. To obtain a uniform seed layer, both the spin-coating and baking processes were repeated three times. Next, a growth solution with equimolar amounts of Zn(NO_3_)_2_6H_2_O and C_6_H_12_N_4_ in deionized (DI) water was prepared to grow ZnO nanorods. The growth solution was stirred for 2 h and transferred into beakers, where the seed layer–coated substrates were suspended upside down. The beaker was sealed with aluminum foil and kept on a hotplate at 125 °C for 5 h to grow the ZnO nanorods. To grow Co-doped ZnO nanorods, four batches of the growth solution were prepared. Co(NO_3_)_2_·6H_2_O with 3, 5, 7, and 9 wt% of Zn(NO_3_)_2_.6H_2_O and C_6_H_12_N_4_ were added to the batches, and Co-doped ZnO nanorods were grown under conditions similar to those used for bare ZnO. After the growth, the bare ZnO and Co-doped ZnO nanorods were washed with DI water and purged with N_2_ gas. Finally, the Co-doped ZnO samples were annealed at 150 °C for 4 h to form the CO/CZO heterostructure. The preparation of CO/CZO heterostructure nanorods on PET substrates is illustrated in Fig. 1. Figure 1 reflects that both [Zn(OH)_2_]^2−^ and Co(OH)_2_]^+^ are growth entities and, because of the electrostatic attraction of Co(OH)_2_]^+^ with O polar and [Zn(OH)_2_]^2^ with Zn polar, the favorable growth position for Co(OH)_2_]^+^ and [Zn(OH)_2_]^2^ is O polar (−) and Zn polar (+), respectively. Therefore, [Zn(OH)_2_]^2^ complex with Zn polar promoted c-plan growth and Co(OH)_2_]^+^ complex and the O polar (−) promoted the lateral growth of Co-doped ZnO nanorods, correspondingly, as can be seen in SEM and XRD analysis. Additionally, owing to the annealing of the Co-doped ZnO, at 150 °C, some of the Co-doped atoms transfer into CoO and form CoO clusters on the (1010 and 101̅0) and (1120 and 11̅20) surfaces of ZnO nanorods due to the low binding energy of CoO over these surfaces; thus, CoO decorated ZnO nanorods, as shown in Fig. 1. The experimental procedures involved in the growth of bare ZnO and heterostructure-based nanorods and their implementation in the pH and glucose sensing setup are illustrated in Additional file 1: Figure S1.

### Calibration and test conditions of potentiometric pH electrode and amperometric glucose electrode

Initially, pH buffer electrolyte solutions having pH values ranging from 3 to 6 and from 8 to 12 were prepared by adding 1.0 M HCl and 1.0 M NaOH to a 1 × PBS solution of pH 7 at room temperature, respectively. The pH calibration of each buffer electrolyte was performed with a commercial glass CRISON 2001 pH meter, while the pH meter was calibrated using three commercial buffer solutions of pH 4, 7, and 10. Next, the bare ZnO and CO/CZO heterostructure electrodes were employed as a sensitive layer for pH sensing in a three-electrode system, where the electrodes based on bare ZnO and the CO/CZO heterostructure were used as the working electrode (WE), while a Pt wire and Ag/AgCl were used as the counter electrode (CE) and reference electrode (RE), respectively. The sensing analysis was performed using current–voltage (I–V) measurements under a voltage range of + 1.0 to − 1.0 and a scan rate of 90 mV s^−1^ in PBS solutions. For each sensing layer, the anodic peak potential in the IV curve, at which the faradaic current is maximum, was used to plot the calibration curve to determine the sensitivity of the corresponding sensor. The pH of human fluids and fruit juices was monitored using a similar three-electrode system. In addition, the pH of each bio-fluid and juice was adjusted using 1.0 M HCl and 1.0 M NaOH. Based on I-V measurement, conducted for pH monitoring, the maximum anodic current response was obtained in 1 × PBS solution of pH 7 solution, showing better oxidation ability of the heterostructure. Therefore, its electrochemical performances towards glucose (0.04 mM to 34.85 mM) detection were evaluated in 1 × PBS solution of pH 7 solution using a three-electrode system at the applied potential from 0 to 1 V. The experimental results showed that maximum current change of glucose was obtained at the potential of + 0.48 V, thus; the amperometric I-t measurements were performed at 0.48 V.

## Results and discussion

### Morphological confirmation of Co dopant and CoO clusters in ZnO nanorods

**Fig. 2:**
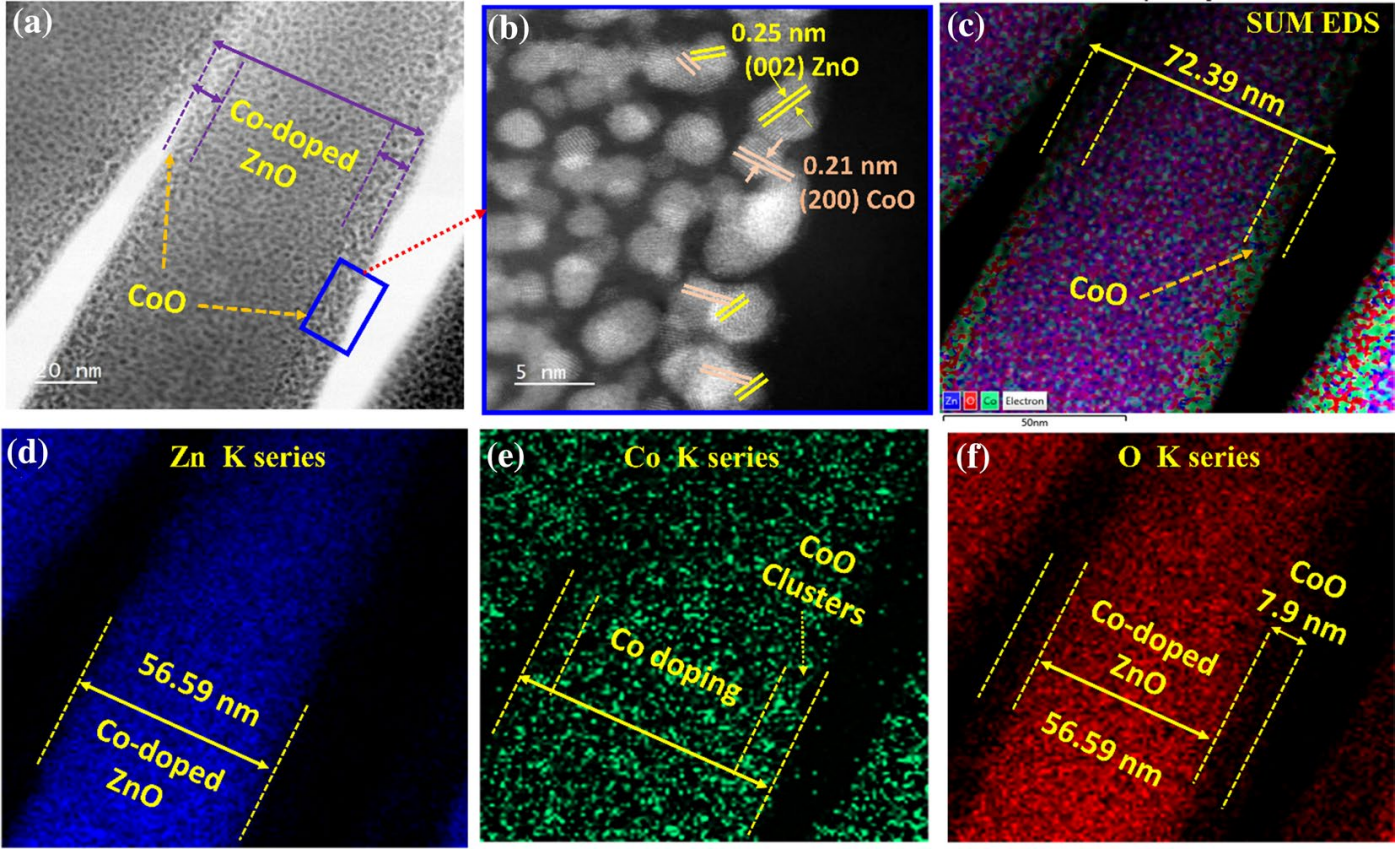
**a** Low-resolution (20 nm) STEM image of CO/CZO heterostructure nanorods and **b** high-resolution (5 nm) STEM image of the area enclosed by a blue rectangle in **a**. **c** Sum EDS mapping image of **a**, which consists of three colors—blue, green, and red—that specify the availability of **d** Zn, **e** Co, and **f** O in the prepared CO/CZO nanorods

Initially, the hexagonal structure and c-plan growth of the bare ZnO and CO/CZO heterostructure nanorods were confirmed by FESEM and HRTEM, as discussed in the supplementary information (SI). Based on the HR-TEM analysis, Co as a dopant was confirmed by observing a smaller d-spacing value (0.25 nm) of ZnO lattices in the CO/CZO heterostructure (Additional file 1: Figure S2i) than bare ZnO (0.26 nm, Additional file 1: Figure S2g), due to a lower ionic radii of Co (0.72 Å) than Zn (0.74 Å), indicating that Co substituted the Zn lattice and vacancy positions and revised the Zn–O and Zn-OH bonds; thus, Co as dopant repaired the defective and relaxed structure of bare ZnO (d_space_ = 0.26 nm), as also shown in sketch (Additional file 1: Figure S5(d–g)). In addition, the Zn K series in the CO/CZO heterostructure exhibited a greater diameter (56.59 nm, Fig. 2g) than the bare ZnO (45.12 nm, Additional file 1: Figure S2g), confirming the existence of the Co dopant and the role of the [Co(OH)_2_]^+^ complex in the promotion of lateral growth (Additional file 1: Figure S4). Moreover, the HR-TEM images (Additional file 1: Figure S2 (h, i) of the CO/CZO heterostructure confirms that CoO clusters are formed, during annealing, on ZnO surfaces owing to the low binding energy of CoO [54, 65]; thus, CoO enhanced the diameter of nanorods. Also, observing the CoO plane (200) adjacent to the ZnO plane (002) (Fig. 2b and Additional file 1: Figure S2i) confirmed the formation of CoO clusters on the ZnO surface. The existence of CoO clusters in Co-doped ZnO nanorods was also confirmed by STEM analysis, as shown in Fig. 2(a, b), where the enlarged view of area enclosed by a blue rectangle depicts CoO clusters on the Co-doped ZnO surfaces. The EDS analysis of Fig. 2a confirms the existence of CoO clusters and the Co dopant in the ZnO structure, as shown in Fig. 2(c–f). The sum EDS images (Fig. 2c) show a diameter of ~ 72.39 nm, of which a diameter of 56.59 nm is attributed to the Zn K series (Fig. 2d) and 15.8 nm is attributed to the Co K series (Fig. 2(c, e)). On the other hand, the O K series is common for both doping and clusters; thus, the O K series (Fig. 2e) demonstrated a diameter similar to that of the sum EDS image. Further, the O K series demonstrated that the density of oxygen is much higher in the ZnO part than that in CoO clusters, reflecting oxygen vacancies in CoO that reduced the crystal size of CoO (as explained in the SI section (Additional file 1: Figure S3f)). The CoO clusters formed over the surface of the nanorods are responsible for the CO/CZO heterostructure formation, while the randomly distributed Co atoms are attributed to the Co doping in the ZnO lattice.

### Structural confirmation of Co dopant and CoO clusters and their influence on reduction of native defects in ZnO nanorods

**Fig. 3:**
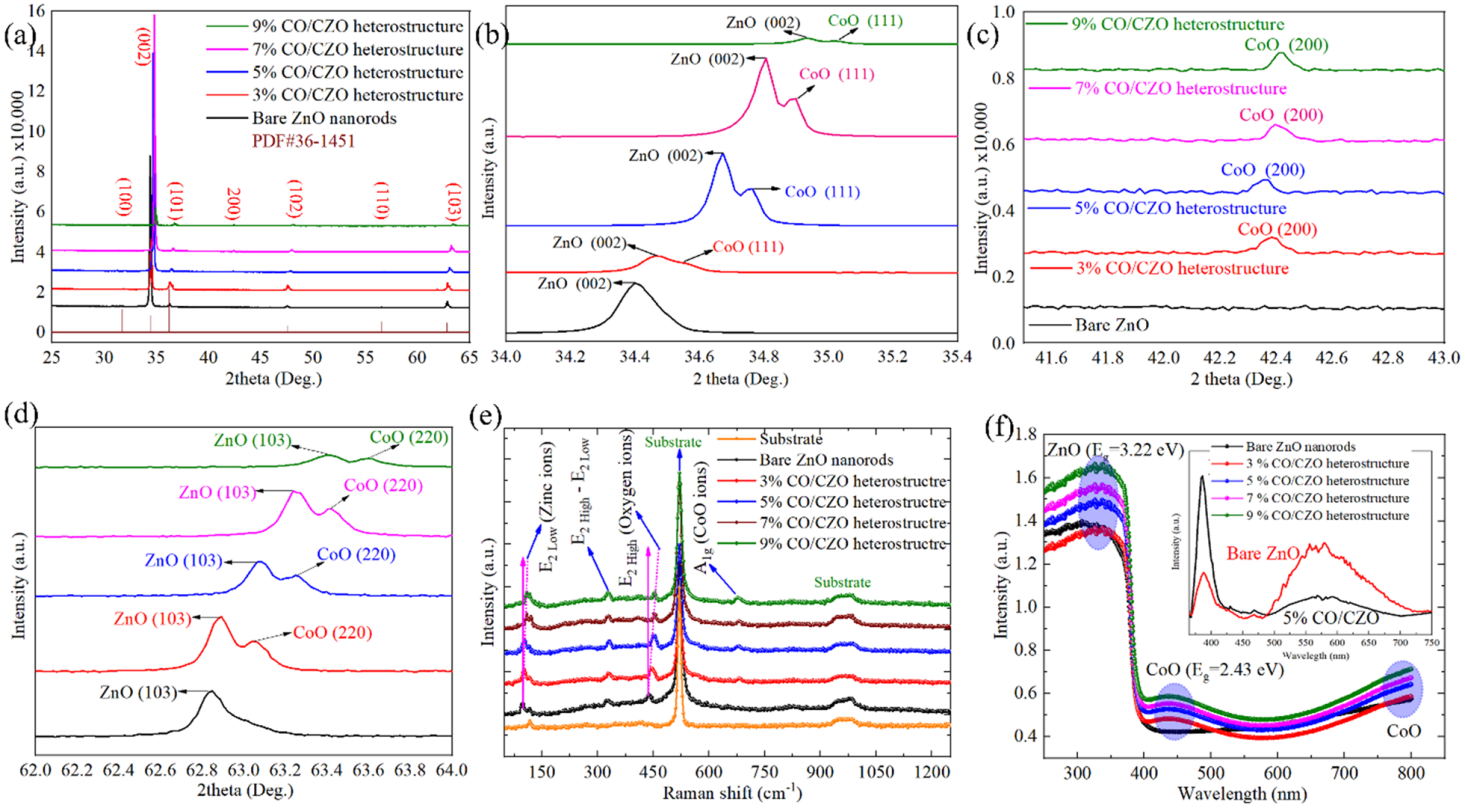
XRD pattern of **a** ZnO nanorods and CO/CZO heterostructure nanorods. Magnified view of **b** plane (002), **c** plane (200), and **d** plane (103). **e** Raman and **f** UV–visible absorption and spectra of bare ZnO and 3%, 5%, 7%, and 7% CO/CZO heterostructure, where the inset shows the spectra of bare and 5% CO/CZO heterostructure

Figure 3a depicts the XRD pattern of bare ZnO and CO/CZO heterostructure, where the (002) peak has a higher intensity than the other five peaks, indicating that the ZnO nanorods grow mainly in the c-plane direction, as detected in the morphological analysis (Additional file 1: Figure S2(a–e)). The magnified view of the (002), (200), and (103) planes showed a secondary phase, which confirmed the existence of CoO in the Co-doped ZnO nanorods, as shown in Fig. 3(b–d). While, Co as dopant was confirmed by obtaining a shift, as shown in Fig. 3(b, d), Additional file 1: Figures S3f and S5(a, b), in several peaks towards a higher 2θ than that of the bare ZnO nanorods. This indicates that the lattice structure of bare ZnO was highly defective owing to the existence of native defects, such as O_v_, Zn_v_, O_in_, and Zn_in_, that enlarged the d-space and created a relaxed and deformed lattice structure (shown by beige areas in Additional file 1: Figure S5(d–f)). Thus, Co as dopant substituted the Zn lattice, O lattice, and vacancy positions and revised the Zn–O and Zn–OH bonding, as result, reduced the defects and repaired the relaxed structure of ZnO. In addition, Fig. 3(b, d) depicted that the peaks (111) and (220) of CoO also shifted toward higher 2θ, demonstrating that the crystal structure of CoO changed due to residual stress caused by point defects, grain boundaries stress, and oxygen vacancies. Since thermal treatment segregated the Co atoms from Co-doped ZnO, forming CoO clusters. The high-energy particles can create an atomic peening effect, which leads to the formation of point defects and oxygen vacancies (non-stoichiometric defects) that have strong effects on the residual stresses. Thus, these factors reduce the bond length and size of the unit cell, resulting in the peaks being shifted towards a larger angle.

Moreover, Raman spectroscopic analysis also confirmed the formation of ZnO and CoO phases and the reduction of defects in CO/CZO, as shown in Fig. 3e. The four peaks in Fig. 3e observed at 99 cm^−1^, 328 cm^−1^, 438 cm^−1^, and 680 cm^−1^ corresponded to E_2-Low_ (zinc ions), E_2-Low_−E_2-High_, E_2-High_ (oxygen ions), and A1_g_ (CoO ions), respectively [58, 66], confirmed the existence of the ZnO and CoO phases. Additionally, compared to the bare ZnO nanorods (Additional file 1: Figure S5i), both the E_2-Low_ (zinc ion) and E_2-High_ (oxygen ion) peaks are shifted toward higher frequencies with increasing Co content in the heterostructure, indicating the reduction of core defects Zn_V_ and O_V_ in the CO/CZO heterostructure [67]. Thus, the Raman spectra and XRD pattern of the CO/CZO heterostructure are in agreement, as the higher side shift in each analysis indicates the reduction of native defects. Furthermore, the UV–visible absorption spectrum (Fig. 3f) shows that the bare ZnO nanorods absorbed high-energy photons and were allowed to transmit all the lower-energy photons. Therefore, the absorption peaks originated at a wavelength of 360.04 nm. However, each CO/CZO-based heterostructure demonstrated additional absorption peaks at wavelengths of 450 nm and 800 nm, confirming the existence of the CoO phase in the CO/CZO heterostructure [68]. The reduction in defect states and trapped carriers at these levels of ZnO was also confirmed by PL spectroscopy analysis, as shown in the inset of Fig. 3f and Additional file 1: Figure S6. For 5% CO/CZO heterostructure, the UV peak reached the maximum intensity, while the defect peak nearly disappeared, confirming that 5% is a suitable addition amount for the significant reduction of defect states in ZnO.

### Improved charge transport and electrochemical activities of ZnO by Co doping and CoO heterostructure

**Fig. 4:**
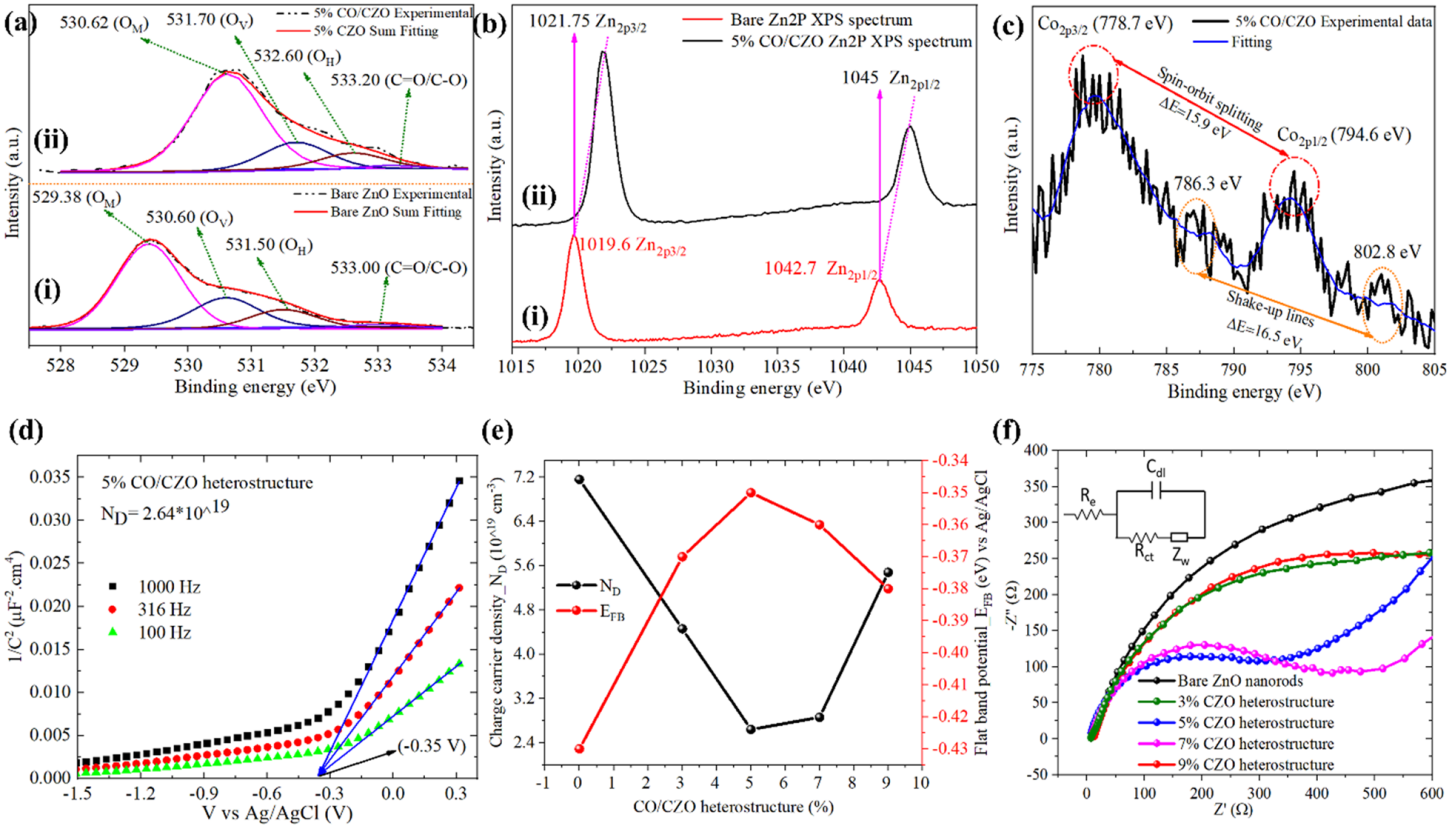
High-resolution core-level XPS **a**-i O1s and **b**-i Zn2p spectra of bare ZnO. **a**-ii, **b**-ii, **c** O1s, Zn2p, and Co2p spectra for the 5% CO/CZO heterostructure, respectively. **d** Mott–Schottky plot for the 5% CO/CZO. **e** Variation in E_FB_ and N_D_ of the bare ZnO and the 3%, 5%, 7%, and 9% CO/CZO heterostructures. **f** EIS plot of the bare ZnO and 3%, 5%, 7%, and 9% CO/CZO heterostructures, where C_dl_, R_e_, R_ct_, and Z_w_ correspond to the double-layer capacitance, electrolyte, charge transfer, and Warburg resistance, respectively, of the equivalent circuit shown in the inset

The enhanced charge density in the space charge region (SCR) and direction of the electric field ($\vec{E})$ at the interface were investigated by XPS analysis, as shown in Fig. 4(a–c). The O1s spectrum of the bare ZnO nanorods (Fig. 4a–i) was deconvoluted into four peaks at binding energies of 529.38 eV, 530.60 eV, 531.50 eV, and 533.0 eV, corresponding to the metal–oxygen (O_M_) bond, oxygen vacancy (O_V_), hydroxyl bond (O_H_), and carboxylic/carbonyl bond (C=O/C–O), respectively [69]. The Zn2p spectra of bare ZnO (Fig. 4b-i) revealed 2 peaks at 1019.6 eV and 1042.7 eV, attributed to Zn2p_3/2_ and Zn2p_1/2_, respectively [70]. Compared to the bare ZnO nanorods, all peaks in the O1s (Fig. 4a-ii) and Zn2p (Fig. 4b-ii) spectra of the CO/CZO heterostructure were shifted toward higher binding energies. These results indicated the interfacial interaction of ZnO and CoO, that allow charge flows from the energy level of ZnO to that of CoO, leading to the generation of $\vec{E}$ directed from ZnO to CoO and increased charge density in the space charge region of the heterojunction. Thus, the charge density decreases in ZnO, necessitating high binding energy to originate the O_M_, O_V_, O_H_, C=O, Zn2p_3/2_, and Zn2p_1/2_ peaks. The Co-2p core levels spectra (Fig. 4c), show a shake-up lines difference of 16.5 eV and spin–orbit splitting difference of 15.9 eV, confirming the presence of the divalent ionic state of Co (Co^2+^) and of the CoO phase in the CO/CZO heterostructure [71, 72].

Moreover, based on the M-S plot (Fig. 4d) discussion mentioned in the SI-section, the estimated E_FB_ values for the bare ZnO nanorods and the 3%, 5%, 7%, and 9% CO/CZO heterostructures were −0.43 eV and −0.37 eV, −0.35 eV, −0.36 eV, and −0.38 eV, respectively, as shown in Fig. 4e. The more positive of E_FB_ of 5% CO/CZO enhanced the conductivity [73], reduced the Schottky barrier height (ɸ_B_) (Additional file 1: Figure S7e); [74, 75], and lowered the overpotential to flatten the E_CB_ band edge (Additional file 1: Figure S7f); thus, promoting the electrochemical activities of 5% CO/CZO. Next, the N_D_ values for bare ZnO nanorods and 3%, 5%, 7%, and 9% CO/CZO heterostructure were calculated to be 7.16 × 10^19^ cm^−3^ and 4.46 × 10^19^ cm^−3^, 2.64 × 10^19^ cm^−3^, 2.86 × 10^19^ cm^−3^, and 5.48 × 10^19^ cm^−3^, respectively (Fig. 4f). The 5% CO/CZO–based electrode exhibited the lowest N_D_ value, confirming that 5% is a suitable addition amount for the significantly reduction of the defective sits in ZnO; thereby, lowering the charge transfer resistance (Fig. 4f) by reducing the Fermi level and ɸ_B_. As the high percentage of Co, i.e., 9%, started to incorporate into the interstitial sites of the lattice, which caused compressive stress, distorted the lattice and enhancing tendency of the Co-3d state formation to trap the free charge carriers; demonstrating higher N_D_ value than other percentage of dopants. Therefore, bare ZnO and 9% CO/CZO demonstrated high R_ct_ value (Fig. 4f). Moreover, EIS analysis (Fig. 4f and Additional file 1: Figure S8) was performed to determine R_ct_ by fitting the semi-circle region with the equivalent circuit (inset in Fig. 4f). The determined R_ct_ values were 950, 756, 317, 388, and 646 Ω for the bare ZnO–based device and 3%, 5%, 7%, and 9% CO/CZO–based devices, respectively. The 5% CO/CZO–based electrode showed the lowest R_ct_ value, attributed to its highest E_FB_ that maximally decreases the ɸ_B_, as sketched in Additional file 1: Figure S7(e, f). All of the discussed analyses confirmed that the Co dopant enhanced N_D_ concentration by reducing the native defects in ZnO, while the CoO cluster lowered R_ct_ by reducing the ɸ_B_ owing to the heterostructure formation.

### Improved pH sensing performances of CO/CZO heterostructure nanorods

**Fig. 5:**
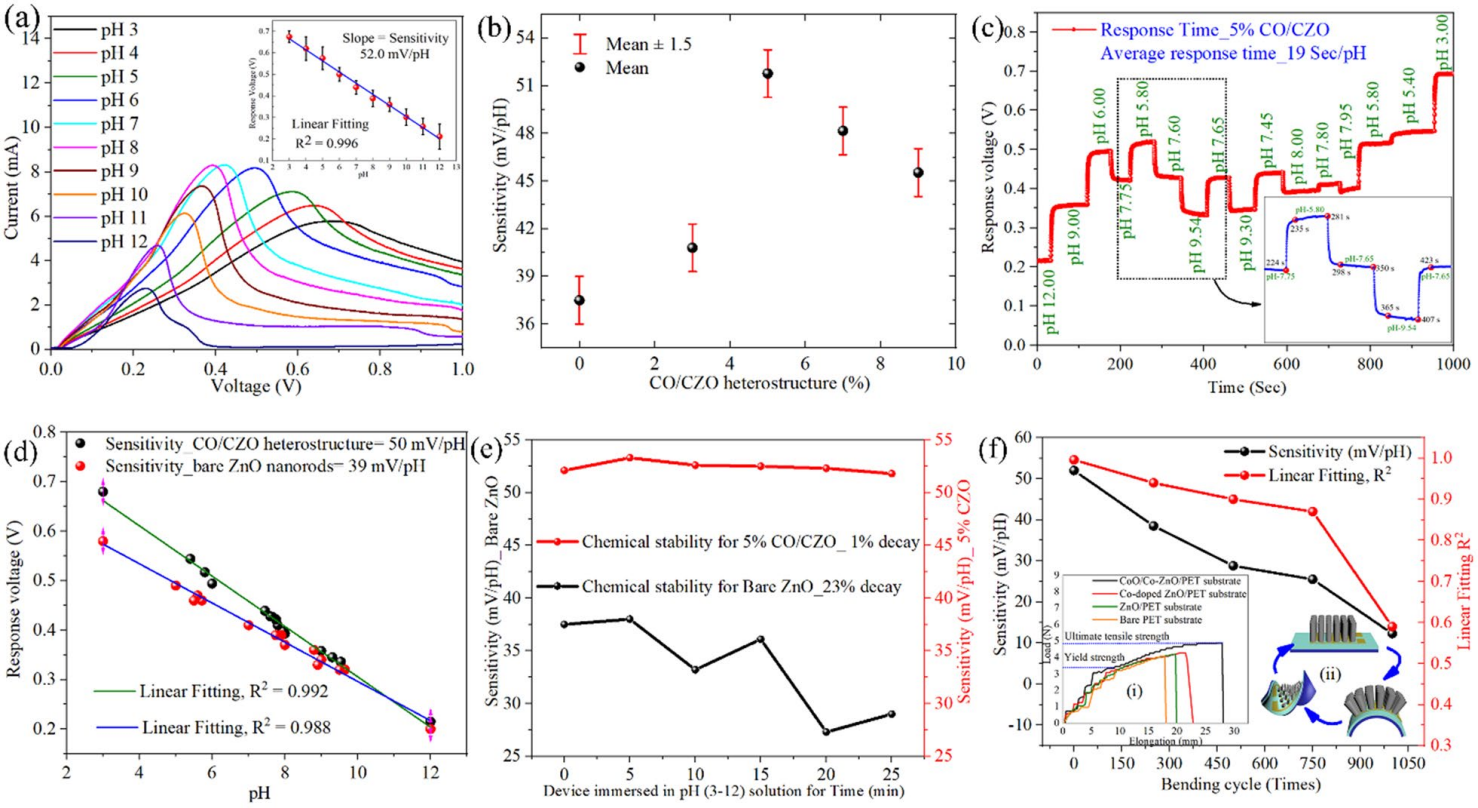
**a** I–V curves for pH sensing performances and (inset in **a**) variation in response voltage of the 5% CO/CZO heterostructure–based electrode. **b** Variation in sensitivity of bare ZnO nanorods and the 3%, 5%, 7%, and 9% CO/CZO heterostructure. **c** Variation in response time of the 5% CO/CZO heterostructure–based device and its corresponding sensitivity determined by the **d** calibration plot. **e** Variation in sensitivity of the bare ZnO-based device and the 5% CO/CZO heterostructure–based device after immersion for 25 min in solutions of different pH values to demonstrate their chemical stability. **f** Variation in sensitivity and linearity of the 5% CO/CZO heterostructure–based device, when it was deformed mechanically as a sketch in inset-(ii), while inset (i) shows stress–strain plot of bare PET, ZnO/PET, Co-doped ZnO/PET, and CO/CZO heterostructure/PET-based samples

To monitor the pH sensing performance, I-V measurements were performed for the 5% CO/CZO heterostructure, as shown in Fig. 5a (and for the bare ZnO, 3%, 7%, and 9% CO/CZO heterostructures, as shown in Additional file 1: Figure S9(a–d), respectively). The potential at the peak of the anodic current was used to plot the calibration curve for the 5% CO/CZO heterostructure (inset in Fig. 5a) and for the bare ZnO, 3%, 7%, and 9% CO/CZO heterostructures (Additional file 1: Figure S10(a–d), respectively). The slope of the calibration plot, which defines the sensitivity of the corresponding sensor [76], was determined to be 37.5, 40.7, 52.0, 48.1, and 46.0 mV/pH for the bare ZnO nanorods and 3%, 5%, 7%, and 9% CO/CZO heterostructures, respectively (Fig. 5b). Next, the response time, defined as the time required for the signal to reach 90% of the equilibrium value while changing the pH of the electrolyte, was determined to be 19 s and 25 s for 5% CO/CZO and bare ZnO, as shown in Fig. 5c and Additional file 1: Figure S11, respectively. The sensitivity of 5% CO/CZO and bare ZnO sensors was determined to be 50 mV/pH and 39 mV/pH, respectively, from the calibration plot of the response time curve (Fig. 5c and Additional file 1: Figure S11, respectively), as shown in Fig. 5d. Moreover, for a fair comparison, sensitivity and response time of 5% Co-doped ZnO nanorods based pH sensor were also determined to be 42.7 mV/pH and 23 s, respectively, as discussed and shown in Additional file 1: Figure S12(a, b). The higher sensitivity and quick response time of the 5% CO/CZO–based sensor compared to the bare ZnO–based and 5% Co-doped ZnO–based sensor can be attributed to the Co doping and CoO clusters formation. The former works as reduction of the density of native defects and improving charges on active site, while the latter generates additional active sites, forms the internal $\vec{E}$ in the heterostructure, higher positive E_FB_, and lower R_CT_.

Moreover, to investigate the chemical stability of bare ZnO and the 5% CO/CZO heterostructure (Fig. 5e), each device was immersed in solutions of different pH values ranging from 3 to 12 for 25 min, as sketched in Additional file 1: Figure S13a. The raw data related to Fig. 5e are presented in Additional file 1: Figures S14(a–j) and S15(a–j) for the bare ZnO and 5% CO/CZO heterostructure, respectively. The sensitivity (Fig. 5e) was determined from the slope of the I-V data, as shown in Additional file 1: Figures S14k and S15k for the bare ZnO and 5% CO/CZO heterostructure, respectively. The results demonstrated that after 25 min, the 5% CO/CZO heterostructure–based device retained 99% of its initial sensitivity, while the bare ZnO–based device maintained only 77% of its initial sensitivity. Additionally, It was confirmed that CO/CZO demonstrated superior mechanical properties, such as yield stress (3.64 N m^−2^), ultimate tensile stress (4.85 N m^−2^), and elongation at break (28 mm) of the ZnO/PET metal oxide–polymer composite, as discussed in the SI section and shown in the inset (i) of Fig. 5f. Therefore, the study was extended to the flexibility analysis of the 5% CO/CZO–based electrode by bending, stretching, and warping it for 1000 cycles, as shown in the inset (ii) of Fig. 5f. The sensing performance was determined after every 250 cycles of bending, stretching, and warping by recording I-V data and observing the change in the anodic peak potential at different pH values of the electrolyte (Additional file 1: Figure S13b). The slope and linearity (R^2^) were determined from Additional file 1: Figure S13b to be 52 mV/pH and 0.996, 38.5 mV/pH and 0.94, 28.8 mV/pH and 0.90, 25.5 mV/pH and 0.87, and 12.3 mV/pH and 0.59 after mechanical deformation for 0, 250, 500, 750, and 1000 cycles, respectively, as shown in Fig. 5f. The obtained results show that the device retained 53% of its initial sensitivity after the first 500 cycles but failed to maintain its sensitivity after 1000 cycles, which reduced the sensitivity and linearity to 12.2 mV/pH and 0.59, respectively. This indicates that mechanical deformation can damage the lattice structure of the CO/CZO heterostructure and the Au pattern underneath the sensing layer, which works as a channel to transfer the charges from the pH electrode to the circuit. The lattice structure of the CO/CZO electrodes may be degraded by multiple cycles of bending, stretching, and other mechanical stresses. However, the pH sensing performance is not likely to be affected in routine applications where significant mechanical stresses are not present.

### Improved glucose sensing performances of CO/CZO heterostructure nanorods

**Fig. 6:**
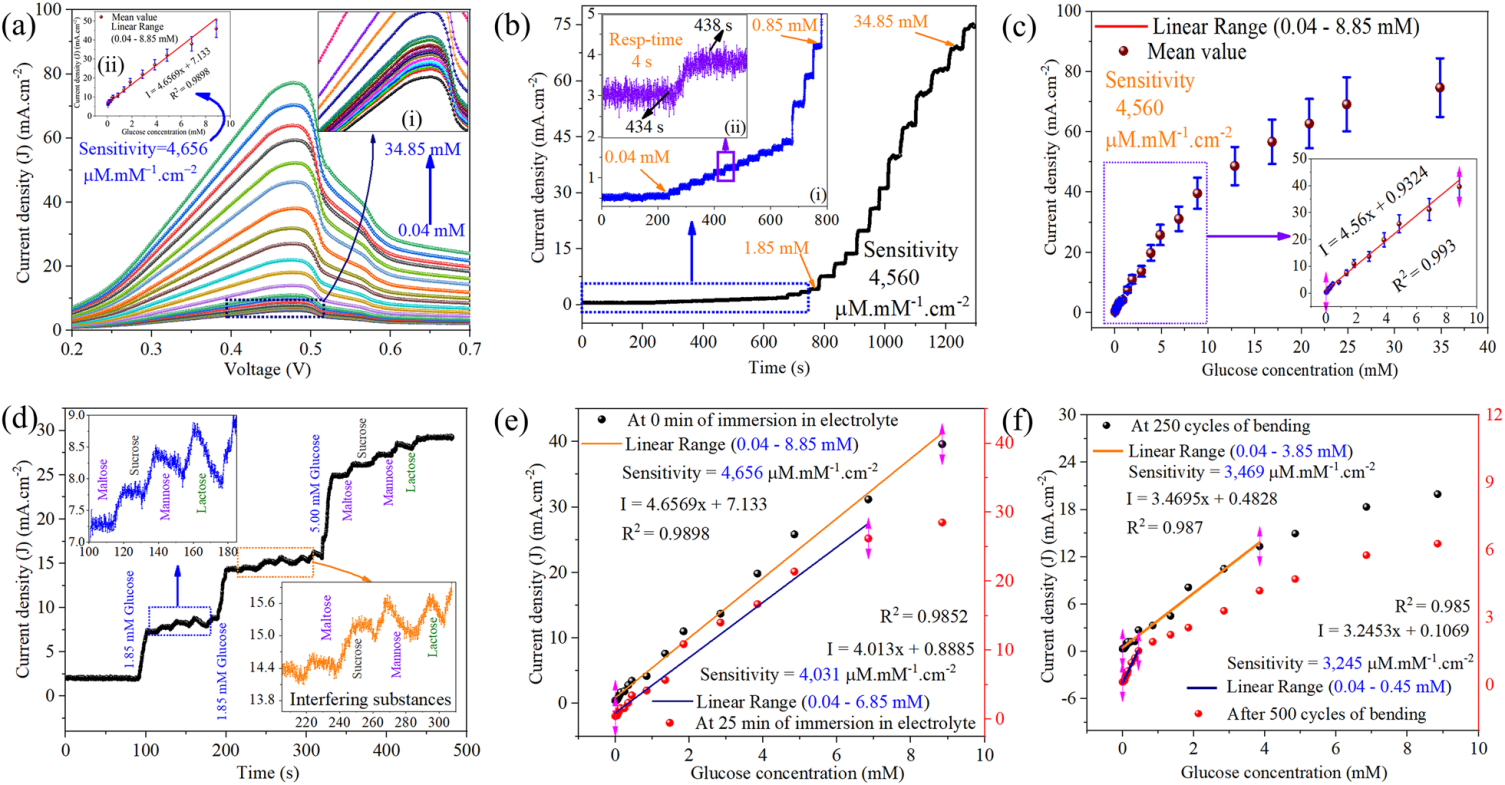
I-V curve for glucose sensing performances of **a** CO/CZO heterostructure, where (inset-i) and inset (ii) show the enlarge of lower concentrated glucose enclosed with dash-blue rectangle and calibration plot of the linear range of 0.04 mM to 8.85 mM, respectively. **b** Amperometric responses to the glucose concentrations varies from 0.04 mM to 34.85 mM. Inset (i) is the enlarge view of the lower concentrated glucose enclosed with dash-blue rectangle and inset (ii) is the sensor response time determined to be 4 s. **c** The measured data and the calibration curve of the sensor’s amperometric response to the glucose concentration, each concentration was repeated four times and the error bars indicate the deviation. Insets plots show the linear relationship between the oxidation peak current and concentration of glucose in the range of 0.04 mM to 8.85 mM and the corresponding regression equation. **d** The amperometric response curve of the sensing electrode to the addition of interfering agents: lactose, maltose, mannose, sucrose, and glucose in the solution. Variation in sensitivity with **e** mechanical deformation of 500 cycles and **f** immersion in electrolyte for 25 mints

Furthermore, the I-V measurement of CO/CZO heterostructure and 5% Co-doped ZnO based electrode was conducted to measure the variation in the peak of the anodic current with different concentrations of glucose, as shown in Fig. 6a and Additional file 1: Figure S17c, respectively. Figure 6a depicts that when the concentration of glucose changed from 0.04 mM to 34.85 mM, the anodic peak current of glucose was linearly related to the glucose concentration over the range of 0.04 mM to 8.85 mM, as shown in the inset (ii) of Fig. 6a. The regression equations was determined to be I/mA = 4.6569x + 7.133c_glucose_/mM (*R*^*2*^ = 0.98) for the linear range, as shown in the calibration curve (inset (ii)) of Fig. 6a). The slope of the calibration plot, which defines the sensitivity of the sensor was determined to be 4,656 μA mM^−1^ cm^−2^ [15]. According to the following equations: LOD = 3.3 × (σ/S), where σ is the standard error of the regression statistics and S is the slope, the limit of detection were calculated to be 0.15 µM [77]. Moreover, the amperometric performance of CO/CZO and bare ZnO nanorods based electrode towards glucose oxidation was examined by I–t measurement under the conditions of continuous stirring of the electrolyte and successive step-wise addition of glucose, as shown in Fig. 6b and Additional file 1: Figure S17a. The calibration plot (Fig. 6c) of Fig. 6b showed that change in current with step-wise addition of glucose was linear till 8.85 mM, as shown in the inset of Fig. 6c. The regression equations and sensitivity were determined for the linear range to be I/mA = 4.56x + 0.9324cglucose/mM (R^2^ = 0.993) and 4560 μA mM^−1^ cm^−2^ correspondingly. Additionally, the response time was determined to 4 s (inset-ii of Fig. 6b). Based on the obtained results (Fig. 6(a–c), it is observed that when the glucose concentration was higher than 8.85 mM the slope of the fitting curve is significantly reduced, indicating a weaker sensitivity of the sensing electrode to the higher glucose concentration. Because, in high concentration, the glucose diffusion rate increased gradually and exceeded its consumption rate at the electrode surface, and as a result, the electrochemical reaction rate is gradually limited by the rate of charge flow. Thus, the response current cannot increase linearly at the higher glucose concentration. For comparative purposes, Table 2 lists the analytical range, sensitivity, and LOD values of CoO, ZnO, or their composite-based electrode materials. It can be seen that our demonstrated low-cost CO/CZO heterostructure-based electrode exhibits a high sensitivity of 4566 μA mM^−1^ cm^−2^, a fast response time of 4 s, and a wide linear response range of 0.04 to 8.85 to glucose among all sensors reported previously as listed in Table 2.

Moreover, the Anti-interference capacity of the CO/CZO heterostructure under similar test conditions as used for I–t measurement was examined as shown in Fig. 6d. Various concentration i.e. 1.85, 2.85, and 5 mM, of glucose and interfering substances were injected at different stage of the analysis. Figure 6d depicts that the response current obtained for 5 mM concentration of the interfering substances is almost negligible than that obtained for 1.85 mM of glucose. Therefore, all the results demonstrated that the present flexible non-enzymatic glucose sensor can possess a good selectivity in the case of actual glucose detection. Moreover, the chemical stability (Fig. 6e) of 5% CO/CZO heterostructure-based sensor was investigated, when it was immersed in PBS solution (pH-7) for 25 min. The chemical stability analysis demonstrated that CO/CZO heterostructure reduced its sensitivity (4031 μA mM^−1^ cm^−2^) by 13% of its initial value (4566 μA mM^−1^ cm^−2^) and linearity range to 6.85 mM from 8.85 mM, as shown in Fig. 6e. Likewise, its flexibility performance was examined, when it was deformed mechanically for 500 cycles (inset-ii of Fig. 5f). Figure 6e shows that CO/CZO heterostructure retained 74% (3469 μA mM^−1^ cm^−2^) and 69% (3245 μA mM^−1^ cm^−2^) sensitivity of its initial value (4566 μA mM^−1^ cm^−2^) after 250 and 500 cycles of bending, respectively, while its linearity range reduced to 3.85 mM and 0.45 mM, correspondingly. The excellent chemical stability and flexibility performances are attributed to the presence of CoO clusters Co-dopant, which helps to protect ZnO/PET from chemicals, moisture, and mechanical degradation [78–81].

### Process of improved pH and glucose sensing performances of CO/CZO heterostructure nanorods

**Fig. 7:**
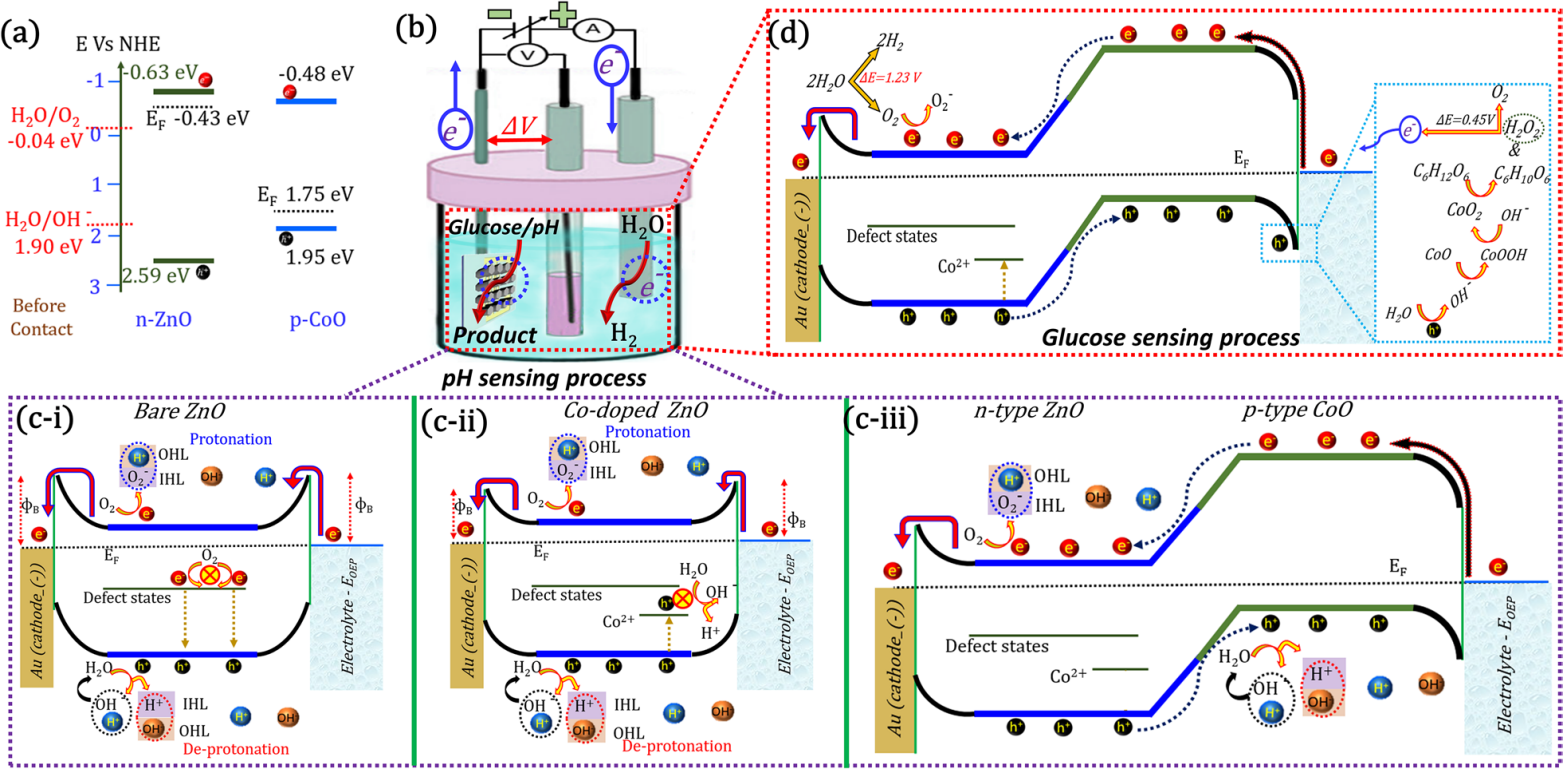
Schematic illustration of energy bands of ZnO and CoO **a** before contact, **b** setup used for glucose/pH sensing, illustration of the process involved in **c** (i–iii) pH and **d** glucose detection

Figure 7 illustrates the process by which the CO/CZO heterostructure shows an improved pH sensing performance compared to bare ZnO nanorods. As the E_CB_ of n-type materials is ~ 0.2 eV higher than the E_FB_; thus, the E_CB_ of bare ZnO can be estimated to be −0.63 eV vs. NHE [82, 83]. According to the empirical formula $E_{CB} = E_{VB} - E_{g}$, the E_VB_ of the bare ZnO can be calculated to be 2.59 eV, as the band gap (E_g_) of ZnO is 3.22 eV, determined by the UV–visible absorption spectra (Fig. 3f) [84]. Similarly, the E_g_ of CoO is determined to be 2.43 eV (Fig. 3f), which is in agreement with a previous study [68], where E_VB_ was determined to be 1.95 eV. The E_CB_ for CoO is calculated to be −0.48 eV. As the E_CB_ of ZnO is more negative than the redox potential of H_2_O/^·^O_2_^−^ (−0.04 eV vs. NHE) (Fig. 7a) [85] therefore, during applied voltage in the electrochemical sensors’ setup (Fig. 7b), charges are transferred from the E_OEP_ to ZnO, where the bare ZnO exhibiting a larger barrier height, lowering R_ct_ value (Fig. 4f). Next, ^·^O_2_^−^ can be produced by the reaction between the free electrons in the E_CB_ of ZnO and O_2_ molecules in the electrolyte, (Fig. 7c(i–iii)). In low pH solution, the O-polar (terminated surface) of ZnO donates electrons to the O_2_ species in the solution that produces an ·O_2_^−^ layer on the oxide surface, namely, an inner Helmholtz layer (IHL), which protonate H^+^ ions (outer Helmholtz layer (OHL)) in the neighboring solution (blue dashed circle in (Fig. 7c(i–iii)). Similarly, the E_VB_ potential of ZnO is more positive than that of ·OH^−^ (1.90 eV vs. NHE) [86], during applied voltage, ·OH^−^ and H^+^ can be produced by the reaction between the holes in the E_VB_ and the H_2_O molecules in the neighboring aqueous solution. In a high pH solution, the resultant product (H^+^) produces an IHL on the ZnO surface, which serves as an electron acceptor for the deprotonation of hydroxyl ions (OHL) in the neighboring solution (red dashed circle in (Fig. 7c(i–iii))). The second entity of the resultant product (OH^−^) combines with H^+^ ions in the solution to reproduce the H_2_O molecule, (black dashed circle in Fig. 7c(i–iii)). The pH sensor relies on the amount of charge accumulated between the OHL and IHL because these surface charges reflect the concentration of H^+^ and/or OH^−^ ions in a solution by changing the voltage of the reference electrode. Also, the total current is proportional to the amount of charge accumulated in the IHL compared to that in the OHL. The accumulation of more charge in the IHL enhances the protonation or deprotonation process that produces a large change in the surface potential as well as in the voltage of the reference electrode per pH value, increasing the sensitivity of the sensors. According to the Nernst equation (Eq. 1) [18], the change in the voltage of the reference electrode is always less than 60 mV per pH:

1 $$E = E^{o} - 2.303 \frac{RT}{F} \times pH = E^{o} - 0.05916 pH.$$

where E, E°, R, T, and F is the measured potential difference, reference electrode potential, gas constant, absolute temperature, and Faraday constant, correspondingly. During applied voltage, owing to the lower band-edge potential of defect levels in ZnO, the charges are trapped in these levels which are then unable to generate ^·^O2^─^ and ^·^OH^─^ radicals, thus limiting their pH sensing properties (Fig. 7c(i)). To minimize these defects in ZnO Co was used as dopant, lowering the R_ct_ value by reducing the ɸ_B_ (Fig. 7c(ii) and Fig. 4f). However, Co as dopant generates an additional Co-3d state at less positive potential (1.19 eV) than the reduction potential, which trapped the carriers and limits their electrochemical activity towards OH^─^ ions in a solution. Therefore, to minimize the trapping probability of charge carriers in the Co-3d state, the Co-doped sample was annealed to induce the CO/CZO heterostructure, as shown in (Fig. 7c(iii)). The CO/CZO heterostructure exhibited an internal $\overrightarrow { E}$, during applied voltage, which provides the necessary driving force for steering the free electrons in the E_CB_ of ZnO from the E_CB_ of CoO and holes from the E_VB_ of ZnO to E_VB_ of CoO. Additionally, CO/CZO heterostructure creates the O-Co-Zn bond, worked as a direct charge-transfer channel between CoO and ZnO, further accelerating the charge transfer process. Owing to the above factors, lower ɸ_B_, heterostructure formation, internal $\overrightarrow { E}$ and the O-Co-Zn bond formation, the hole and electron density in the E_VB_ of CoO and E_CB_ of ZnO is greater than that in the E_VB_ and E_CB_ of bare ZnO. Therefore, the CO/CZO heterostructure enhances the protonation and deprotonation activity, demonstrating a higher pH sensitivity, near to the Nernst limit (52 mV/pH), than bare ZnO based pH sensors and those in previous reports, as listed in Table 1.

**Table 1:** Performance comparison of ZnO-based pH sensor with those reported previously

Sensing layer	pH range	Sensitivity (mV/pH)	Response time (s)	Electrolyte volume	References
ZnO thin film	2–12	38	–	Immersed in electrolyte	[88]
ZnO NRS/NTs	4–12	45.9	–	Immersed in electrolyte	[34]
ZnO/Ta thin film	1.3–12	41.56	–	Immersed in electrolyte	[89]
ZnO thin film	4–12	49.35	–	Immersed in electrolyte	[90]
InGaZnO thin film	3–10	59.2	< 300	Immersed in electrolyte	[91]
ZnO thin film	2–9	43.71	29	5 mL	[26]
ZnO NW	4–12	45.89	< 0.5	5 mL	[3]
Al-doped ZnO NW	2–12	50.3	< 0.5	5 mL	[3]
In−Ga−ZnO nanoparticle/Si Nanowire	2–10	50	60	Immersed in electrolyte	[92]
ZnO nanotube	4–12	45.9	300	Immersed in electrolyte	[34]
ZnO nanorod	4–12	28.4	300	Immersed in electrolyte	[34]
Passivated intrinsic zinc-oxide nanorod	4–12	44.01	–	Immersed in electrolyte	[90]
ZnO thin film	2–9	43.71	29	5 ml	[26]
CO/CZO heterostructure	3–12	52.2	19	Immersed in electrolyte	This work
5% Co-doped ZnO	3–12	42.7	22–23	Immersed in electrolyte	This work

Moreover, owing to the advantage of lower ɸ_B_, heterostructure formation, internal $\overrightarrow { E}$ and the O–Co–Zn bond formation, the CO/CZO heterostructure has also shown improved glucose-sensing performances, as listed in Table 2. The process of the improved glucose sensing performance is illustrated in Fig. 7(b, d), where, prior to the injection of glucose, ·O^2─^ is produced by the reaction that occurs between the electrons in the E_CB_ of ZnO and the O_2_ species in the solution, resulting in the current of the oxidation peak is generated (Fig. 6a). It is that Zn (II) is oxidized to Zn (III) (reaction SI 11). Meanwhile, owing to heterostructure, applied potential in forward bias, and internal $\overrightarrow { E}$ steered holes from the E_VB_ of ZnO to the E_VB_ of CoO, which oxidizes the H_2_O molecule to generate OH^−^ radicle. The OH^−^ radicles are then reacted with CoO to produce CoOOH and release an electron, where the free-electron reduced the H_2_O molecule to produce OH^−^ radicle (reactions SI 12 and SI 13). The produced OH^−^ radicles combined with CoOOH species, generating CoO_2_ species, H_2_O molecules and releasing an electron (reaction SI 14), which reverted the Zn (III) to Zn (II) (reaction SI 15). Next, when glucose is added, the glucose molecules (C_6_H_12_O_6_) are catalytically oxidized by CoO_2_ species to gluconolactone (C_6_H_10_O_6_) and H_2_O_2_ (reaction SI 16). Finally, the H_2_O_2_ oxidizes to produce H_2_O molecules, O_2_ molecules and release two electrons (reaction SI 17). The released electrons are transferred to the circuit and increased the current of oxidation peak which is then received by the H_2_O molecules on the cathode side, while the O_2_ molecules restart the process.

**Table 2:** Performance comparison of CO/CZO based glucose sensors with ZnO, CoO and their heterostructure either with a metal or a metal-oxide reported previously

Sample	Linear range (mM)	Sensitivity (µA mM^−1^ cm^−2^)	The detection limit (µM)	Response time (s)	References
CO/CZO	0.04 –8.85	4565	0.15	~ 4	This study
5% Co-doped ZnO	0.04–8.85	3864	0.30	–	This study
Bare ZnO	0.0	2565	0.33	~ 10	This study
ZnO/CC	0.001–1.45	4792	0.43	3	[93]
CuO-ZnO	Upto 8.45	2961	0.4	–	[94]
ZnO nanocombs	0.02–4.5	1533	20	–	[95]
Au-ZnO NRs	0.001–15	4416	0.12	< 2	[96]
ZnO nanorods	0.6–1.4	1091	0.22	–	[97]
Porous ZnO-CuO hierarchical nanocomposites/FTO	0.00047–1.60	3066	0.21	1.2	[98]
Co_3_O_4_ nanosheets	Up to 0.31	12,970	0.058	< 10	[99]
ZnO–CoO/rGO	0.01–11.20	168.7	1.3	–	[100]
Co_3_O_4_/ZnO p–n junction	0.01–5	116.64	1.38	2	[101]

### Application of CO/CZO heterostructure–based pH and glucose electrode to human fluids and fruit juices

**Fig. 8:**
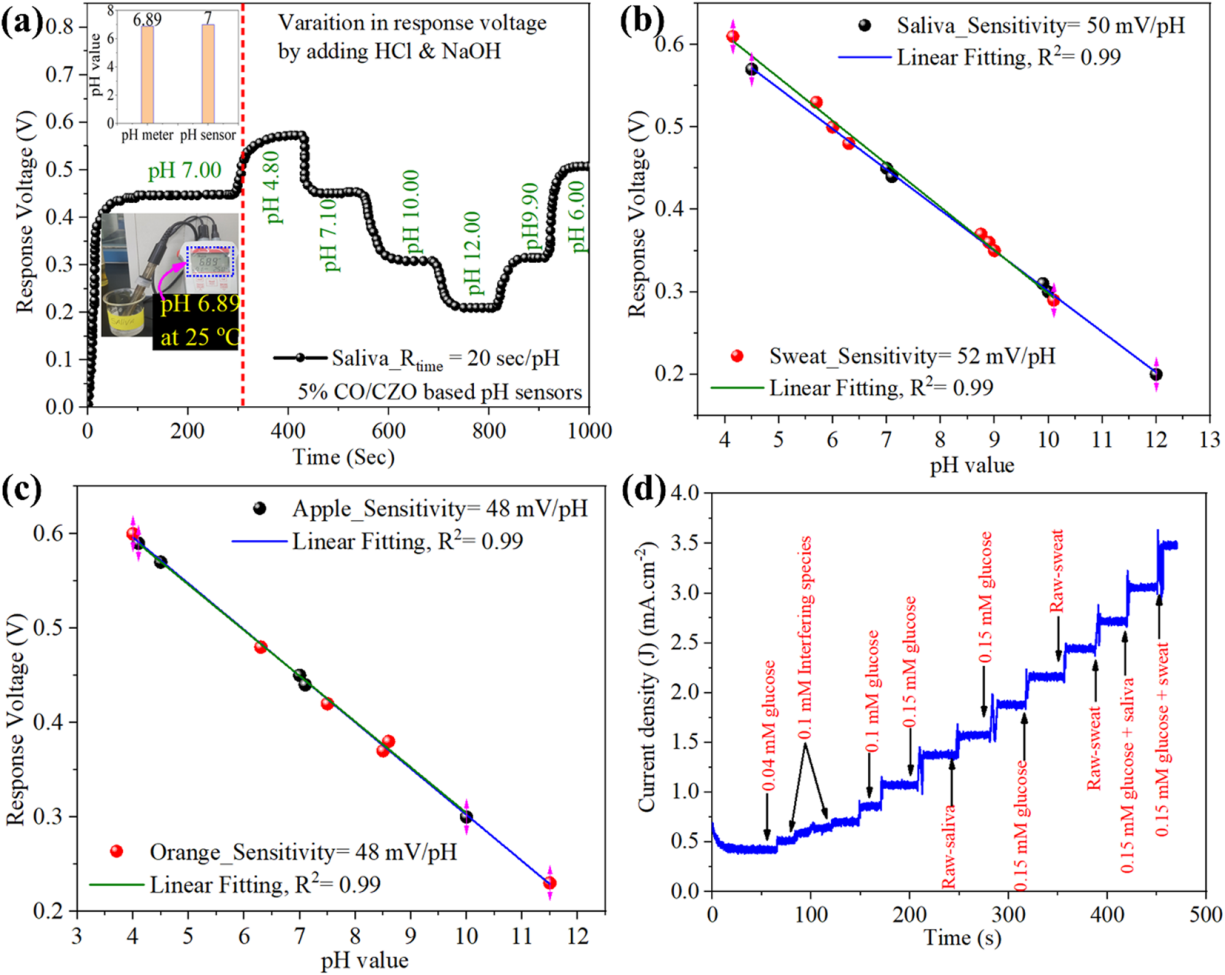
**a** Real-time monitoring results of pH in human saliva (insets: (above) bar graph comparing the pH results obtained by pH meter and pH sensor. Sensitivity response of the CO/CZO based device when immersed in **b** saliva and sweat fluids and **c** apple and orange and changing their pH with HCl and NaOH. **d** Amperometric I-t plot of CO/CZO based electrodes towards raw and glucose added human fluid

Finally, the practical significance of the fabricated CO/CZO-based pH sensor was demonstrated by employing it in real-time applications and monitoring the pH in saliva (Fig. 8a), sweat (Additional file 1: Figure S16a), and fruit juices, namely, apple, orange, lemon, and grape juice, as shown in Additional file 1: Figure S16(b–e), respectively. The results were compared with the results obtained using a commercial glass CRISON 2001 pH-meter, as shown in the photograph in the inset of Fig. 8a and Additional file 1: Figure S16(a–e). The bar graph in the inset of Fig. 8a and Additional file 1: Figure S16(a–e) demonstrate that the pH values of the body fluids and fruit juices measured by the developed pH sensor were very close to those measured by the pH meter. The variation in the response voltage was measured by adding 1.0 M HCl and 1.0 M NaOH solutions to the body fluids (Fig. 8b) and fruit juices (Fig. 8c Additional file 1: Figure S16f). In the plot of response voltage vs. pH, the pH sensors showed sensitivity responses of 50, 52, 48, 48, 58, and 52 mV/pH for saliva, sweat, apple, orange, lemon, and grape samples, as shown in Fig. 8b, c, and Additional file 1: Figure S16f. Thus, it can be concluded that the fabricated sensor is highly reproducible and unaffected by interference ion species when applied to healthy human fluid or fresh juice samples. Moreover, the practical application of CO/CZO heterostructure was further extended to saliva- and sweat-glucose-based sensing analysis. The concentration of glucose in human fluids, such as saliva and sweat, is in the range of 0.02 mM to 0.2 mM and 0.2 mM to 0.6 mM, respectively; thus, these two human fluids can be employed for glucose detection [9, 87]. Since the LOD of our constructed electrode is 0.15 mM, therefore, it can be worked for non-invasive glucose sensing using human fluids. To conduct this experiment, 150 µM concentrated glucose was added to the raw saliva and sweat samples and measure the amperometric I-t response for each raw-fluid and glucose-added fluid-based sample, as shown in Fig. 8d. Figure 8d depicts that CO/CZO based electrode demonstrated a rapid and high amperometric response towards each raw-fluid and glucose-added fluid-based sample compared to the interfering substance, demonstrating a powerful potential for glucose detection with a high degree of accuracy.

## Conclusion

The present study demonstrated that Co doping in ZnO can induce a CoO phase upon annealing at a temperature of ≥ 150 °C, resolving the conflict in previous studies regarding the favorable conditions for the formation of the Co or CoO phase in ZnO. The induced CoO phase forms a p-n heterostructure with Co-doped ZnO over a PET substrate, overcoming the challenges of metal-oxide integration with low-melting-point polymeric substrates for flexible electronic systems. Additionally, in the CO/CZO heterostructure, the Co dopant decreased the N_D_ at the non-active sites, improved yield stress of ZnO, and lower the R_ct_ from electrolyte to electrode by reducing the ɸ_B_, while CoO helped to improve the corrosion resistance and fatigue life of the ZnO/PET metal oxide–polymer composite by binding together the reinforcement phases, including the PET fiber, ZnO nanorods, Co atoms, and CoO nanoparticles, in the CO/CZO/PET composite and protecting the composite from the chemicals, moisture, and mechanical degradation. The CO/CZO heterostructure demonstrated high pH sensitivity (52 mV/pH), high chemical stability (1% degradation in 25 min), high flexibility (retained 52% of its initial sensitivity after mechanical deformation for 500 cycles), and quick response (19 s) when applied as a pH electrode in PBS solutions of different pH values (ranging from 3 to 12). In addition, the fabricated CO/CZO-based sensor is highly reproducible and unaffected by interference ion species when applied to healthy human fluid or fresh juice samples. Moreover, the developed CO/CZO-based electrode demonstrated high sensitivity (4,656 µM mM^−1^ cm^−2^), low limit of detection (0.15 µM), a broad linear range (0.04 mM to 8.85 mM), and good selectivity against various interference substances towards glucose-sensing, representing an influential prospective for glucose detection with a high degree of accuracy. Therefore, the CO/CZO heterostructure can fulfill the demand for inexpensive, portable, low-cost, and precise miniature pH and non-enzymatic glucose sensors that allow easy integration with wearable devices for use in healthcare. Our study is a significant step toward the development of flexible, sensitive, and robust miniature pH and non-enzymatic glucose sensors using a metal oxide grown at low temperatures. The methods used in this study for fabricating the CO/CZO heterostructure can be applied to other metal-oxide combinations to create electronic sensors with desirable properties for various applications.

## Supplementary Information

**Additional file 1.** A Dual-functional flexible sensor based on defects-free Co-doped ZnO nanorods decorated with CoO clusters towards pH and glucose monitoring of fruit juices and human fluids.

## Data Availability

The authors have no data to share since all data are shown in the submitted manuscript.
