# Supplementary material for: A dual-functional flexible sensor based on defects-free Co-doped ZnO nanorods decorated with CoO clusters towards pH and glucose monitoring of fruit juices and human fluids
Source: Nano Converg. 2022 Mar 22;9:14. doi: 10.1186/s40580-022-00305-x (PMC8941038; doi:10.1186/s40580-022-00305-x)
Supplement: Supplementary file 1 — Additional file 1. A Dual-functional flexible sensor based on defects-free Co-doped ZnO nanorods decorated with CoO clusters towards pH and glucose monitoring of fruit juices and human fluids. [file 40580_2022_305_MOESM1_ESM.docx]

**Electronic Supplementary Information**

**A Dual-functional flexible sensor based on defects-free Co-doped ZnO nanorods decorated with CoO clusters towards pH and glucose monitoring of fruit juices and human fluids**

Muhammad Hilal and Woochul Yang*

Department of physics, Dongguk University, Seoul 04620, Republic of Korea

*Corresponding author: [**wyang@dongguk.edu**](mailto:wyang@dongguk.edu)

- 1. **Experimental details**
  2. **Reagents and solutions**

Zinc acetate dehydrate [Zn(CH_3_COO)_2_·2H_2_O], hexamethylenetetramine (C_6_H_12_N_4_), zinc nitrate hexahydrate [Zn(NO_3_)_2_6H_2_O], cobalt (II) nitrate hexahydrate [Co(NO_3_)_2_·6 H_2_O], 1-propanol, phosphate-buffered saline (PBS), buffer solutions (pH 4, 7, and 10), gold (Au) pellets, and polyethylene terephthalate (PET) sheets were obtained from Sigma Aldrich and used without further purification.

- 1. **Characterization of ZnO and CO/CZO heterostructure nanorods**

After the successful growth of the ZnO and CO/CZO heterostructure nanorods on flexible PET substrates, the morphological analysis along with elemental composition was performed using a field-emission scanning electron microscope (FESEM, JEOL-6700s) and high-resolution HR-TEM and STEM (JEOL JEM-ARM200F “NEOARM”) equipped with energy-dispersive X-ray spectroscopy (EDS). The structure’s confirmation of bare ZnO and its heterostructure CoO were examined using XRD equipment (Rigaku Ultima IV) with Cu KR (λ = 1.542 Å) as the incident beam. The XRD scanning was performed at a rate of 0.20 s^-1^ in a 2θ range of 30º–65º. Similarly, Raman spectra were obtained using a DXR Raman microscope with green-lighting excitation (532 nm). The optical properties, including absorbance and photoluminescence (PL), were studied using a V-750 UV-visible spectrophotometer equipped with diffuse reflectance spectra and a fluorescence spectrophotometer (Model: JASCO FP-8600), respectively. The bonding properties and environmental impurities of the elements observed in the bare ZnO nanorods and CO/CZO heterostructure were confirmed using a Kα X-ray photoelectron spectrometer (Thermo VG, UK) equipped with a monochromatic Al X-ray source (Al Kα line: 1486.6 eV). All electrochemical analyses, including electrochemical impedance spectroscopy (EIS), Mott–Schottky plots, pH sensing, and pH response time measurements, were performed using a computer-controlled biologic potentiostat (VSP-300) operated using EC-lab (version 10.40) software. All measurements were performed at room temperature (25 °C ± 1 °C).

**
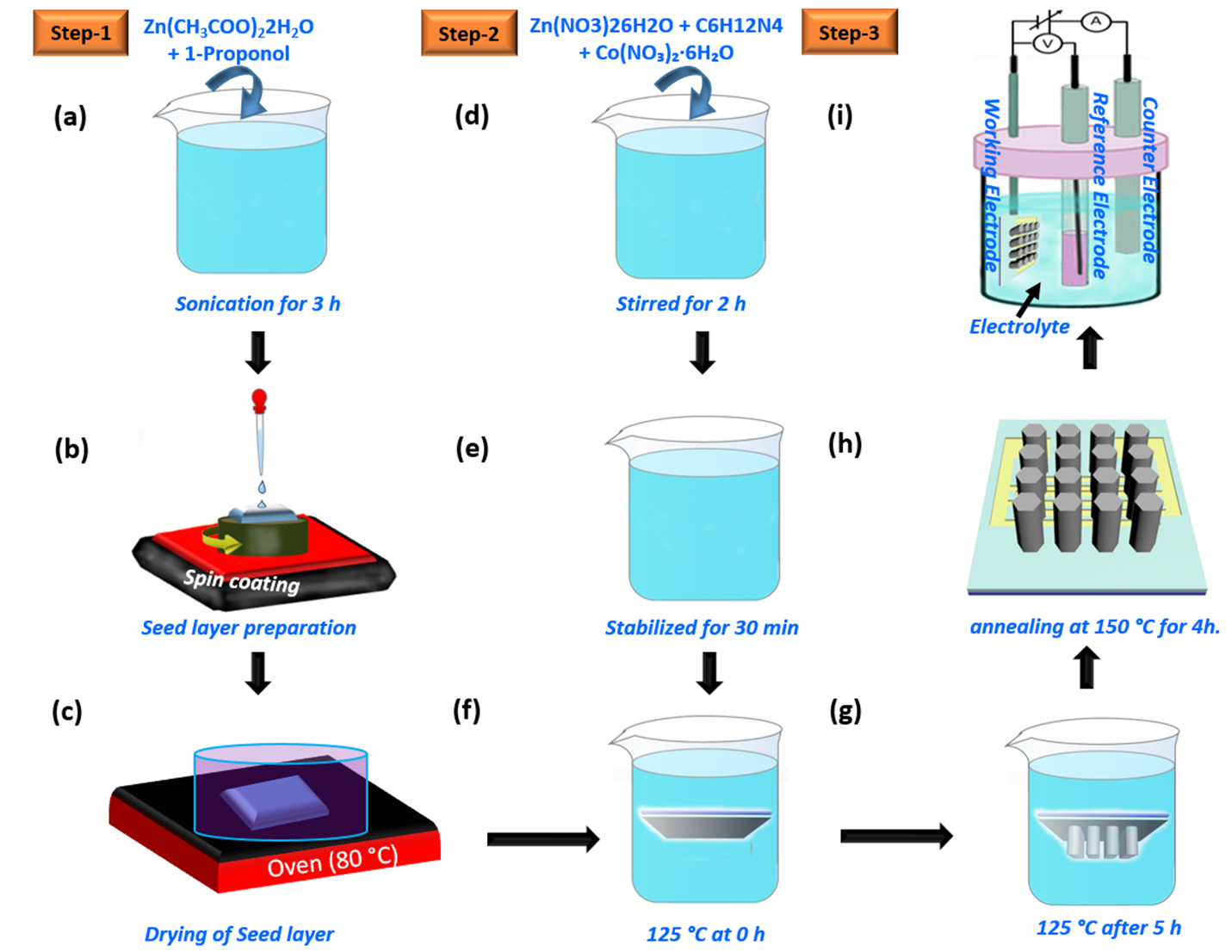
**

**Fig. S1**. Steps involved in the synthesis of CoO-ZnO P-N heterostructure nanorods and in the fabrication of the pH electrode.

1. **Results and discussions**
   1. **Morphological analysis of ZnO and CO/CZO heterostructure nanorods**

The surface morphologies of the bare ZnO, 3%, 7%, and 9% CO/CZO heterostructure nanorods were analyzed using FESEM, as shown in Fig. SI 2 (a-e), respectively. Each top-view image showed a hexagonal structure of the material, with a homogeneous density and relatively unidirectional growth in the c-plane. Additionally, it was observed that Co doping widened the diameters of the nanorods (Fig. SI 2f), indicating the hindrance of vertical growth by Co and promotion of lateral growth. This is because, in bare ZnO, all available hydroxide (OH^-^) in the solution complex with Zn^2+^, thus, enabling [Zn(OH)_2_]^2-^ as the only entity for the ZnO nanorods growth. Therefore, [Zn(OH)_2_]^2-^ ions and the positively charged Zn-polar top surface promoted axial growth in the Zn-polar direction. However, in CO/CZO heterostructure, some amount of the OH^-^ complexed with Co^2+^ and produced [Co(OH)_2_]^+^, enabling both [Zn(OH)_2_]^2-^ and Co(OH)_2_]^+^ as growth entities. Because of the electrostatic attraction between Co(OH)_2_]^+^ and O polar (-), the favorable growth position for Co(OH)_2_]^+^ is O polar (-). A possible mechanism explaining this observation is presented by the diagram shown in the inset of Fig. SI 4. Reactions (1-8) denotes [Zn(OH)_2_]^2-^ complex formation and vertical growth of nanorods, while reaction (9-10) corresponds to [Co(OH)_2_]^+^ complex formation and lateral growth of nanorods. Additionally, owing to the annealing of the Co-doped ZnO some of the Co-doped atoms transfer into CoO and form CoO clusters on the (1010 and 101̅0) and (1120 and 11̅20) surfaces of ZnO nanorods [[1](#_ENREF_1), [2](#_ENREF_2)]; thus, Co doping widened the diameters of the nanorods. The HR-TEM image (Fig. SI 2g) confirmed that the bare ZnO nanorods are single crystals, exhibiting orientation along the (002) plane, a diameter of approximately 45.12 nm, and a d-spacing of 0.26 nm (inset image in Fig. SI 2g).

Moreover, HRTEM analysis and EDS mapping of 5% Co-doped ZnO nanorods-based sample were conducted as shown in Fig. SI 3 (a-e). The obtained results confirmed that Co as a dopant reduces the lattice spacing of ZnO (Fig. SI-3c) and [Co(OH)_2_]^+^ complex promotes the lateral growth (Fig. SI-3e), demonstrating a larger diameter of 63 nm than bare ZnO (45 nm). Further, according to Bragg’s law, reduction of latticed-spacing shifted the 2 theta towards a larger angle. Thereby, in the XRD pattern (Fig. SI-3f), peaks were shifted towards a higher 2θ than that of the bare ZnO nanorods. In addition, when Zn site ion is replaced by smaller ions (Co) the overall volume of ZnO shows shrinking, decreasing the unit cell. Since 9% sample contained a high concentration of doping, thus a greater reduction in the crystalline size can be formed. Small crystallites possess a restricted number of reflection planes, demonstrating less intense and broad XRD peaks (Inset of Fig. SI-3f).

**
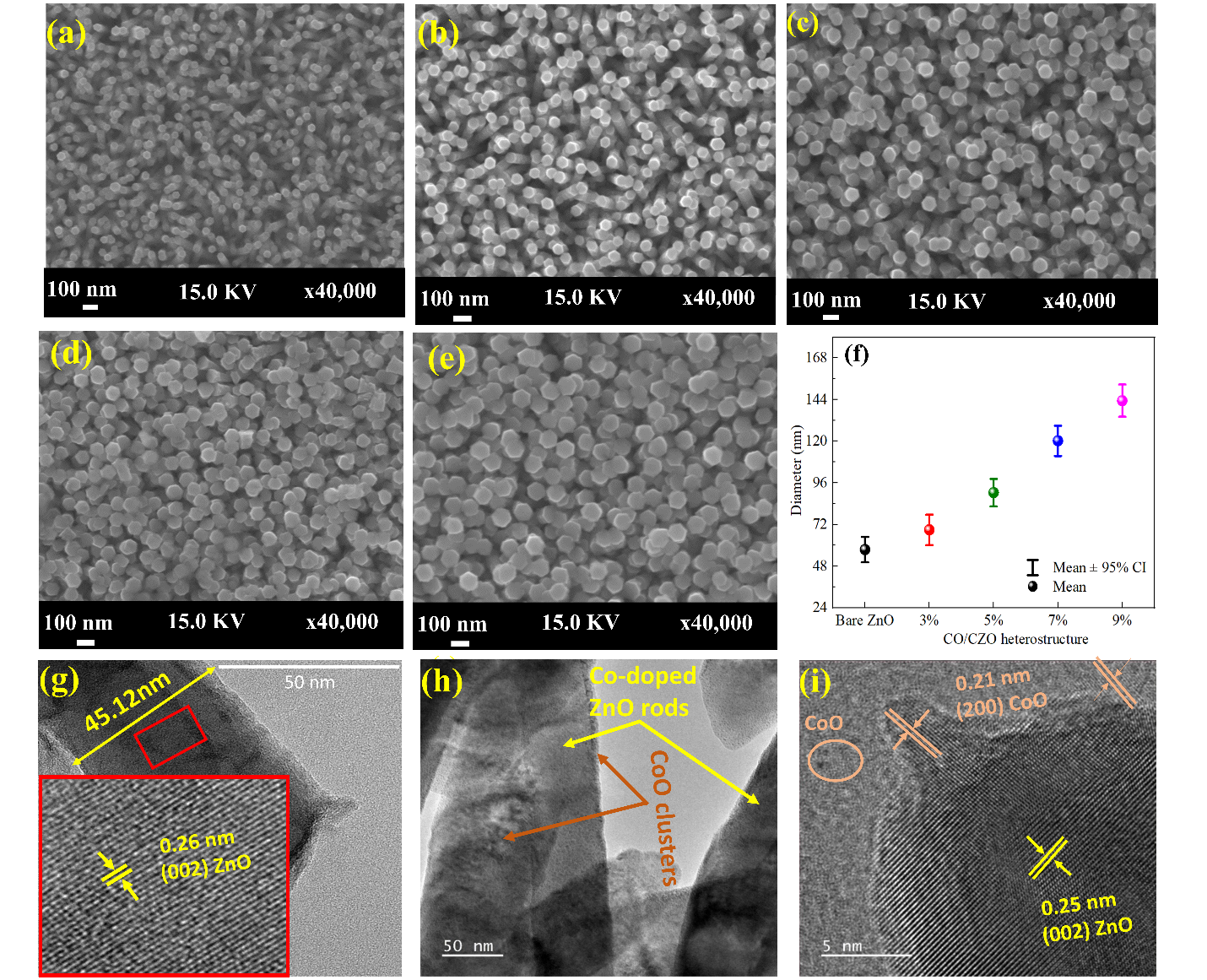
**

**Fig. S2.** Front view of FESEM images of (a)bare ZnO, (b) 3%, (c) 5%, (d) 7%, and (e) 9% CO/CZO heterostructure nanorods. (f) Variation in the diameter of nanorods with Co incorporation and CoO cluster formation. HR-TEM images of (g) bare ZnO and (h, i) CO/CZO heterostructure nanorods.


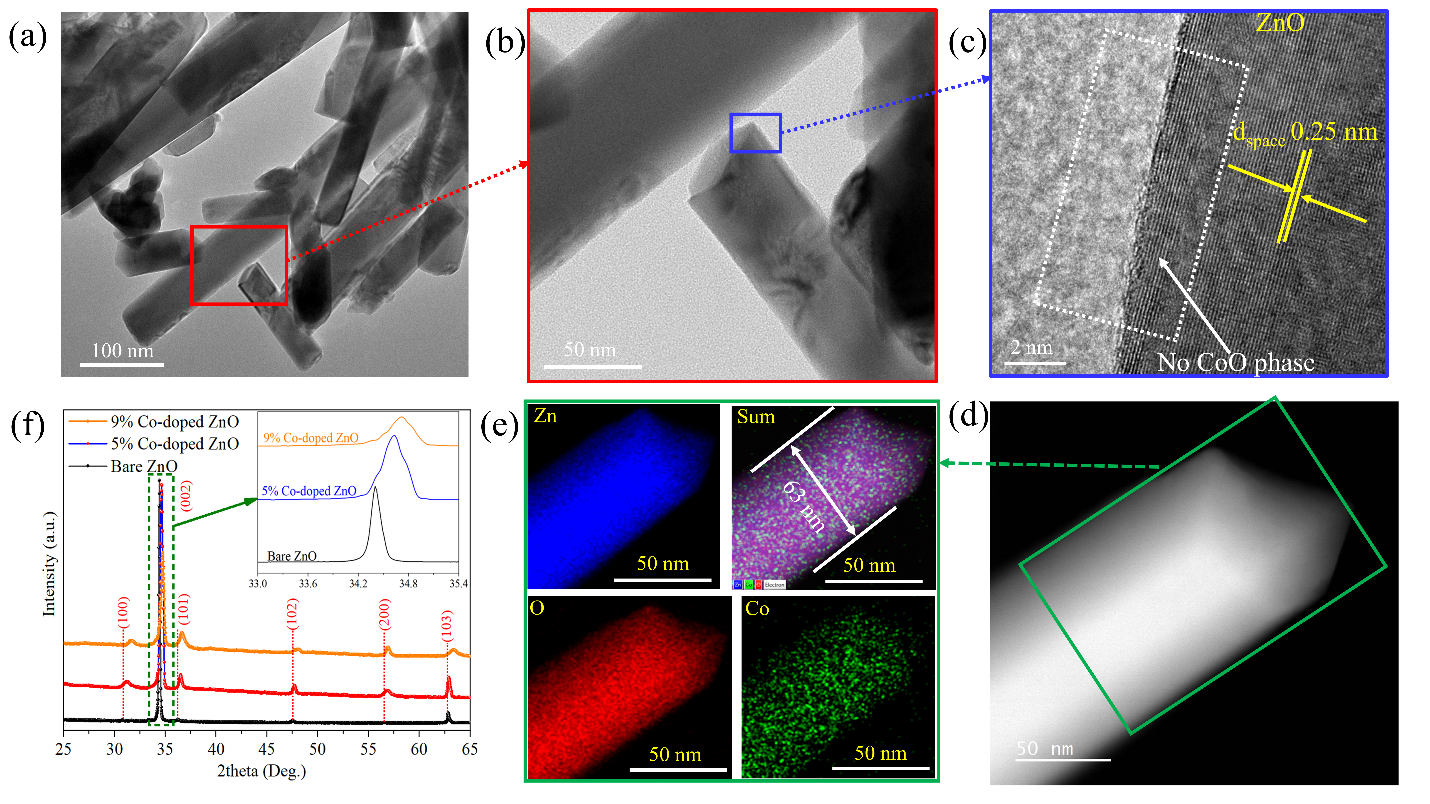


**Fig. S3.** (a-c) HRTEM images of 5% Co-doped ZnO nanorods, (d) HRTEM image used for (e) EDS mapping, which consists of three colors—blue, red, and green—that specify the availability of Zn, O, and Co in the prepared 5% Co-ZnO nanorods. (f) XRD pattern of bare ZnO and 5%, and 9% Co-doped ZnO nanorods.


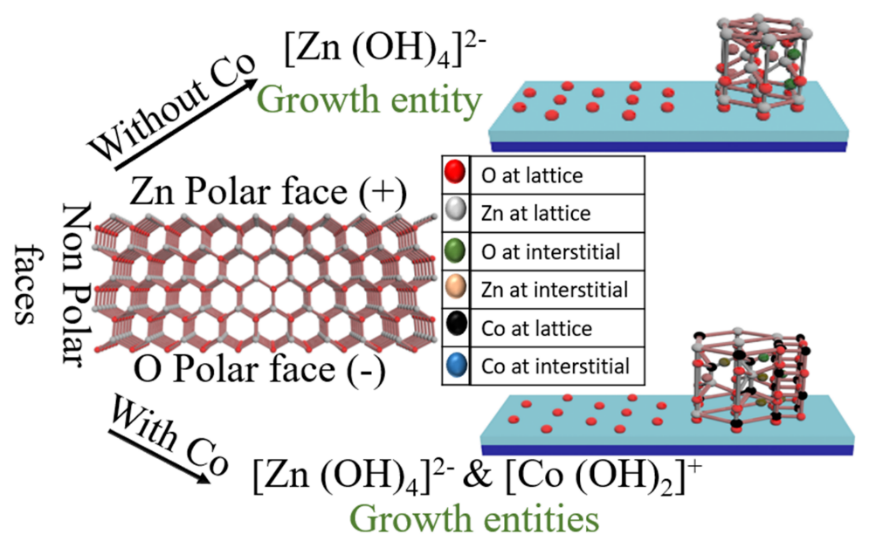


**Fig. S4.** Sketch showing the diameter increase of nanorods with Co incorporation and the formation of CoO clusters.

(CH_2_)_6_N_4_ + 6H_2_O → 4NH_3_ + 6HCHO ---------------------- (1)

NH_3_ + H_2_O^+^ → NH_4_^+^ + HO^-^ ---------------------------------- (2)

Zn(NO_3_)_2_ → Zn^2+^ + 2NO^-^_3_ ---------------------------------(3)

Zn^2+^ + HO^-^ → Zn(OH)^+^ ----------------------------------- (4)

Zn(OH)^+^ + HO^-^ → Zn(OH)_2_ ------------------------------ (5)

Zn(OH)_2_+ HO^-^ → Zn(OH)^-^_3_ ------------------------------ (6)

Zn(OH)^-^_3_ +2HO^-^ → Zn(OH)^2-^_4_ --------------------------- (7)

Zn(OH)^2-^_4_ → ZnO + H_2_O+ 2OH^-^ --------------------------(8)

Co^2+^ + 6NH_3_ → Co(NH_3_)^2+^ ------------------------------(9)

Co(NH_3_)^2+^ + HO^-^ → Co-complex----------------------- (10)

- 1. **Structural analysis of ZnO and CO/CZO heterostructure nanorods**

The XRD pattern (Fig. 3a) contained six peaks at diffraction angles (2θ) of 30.79°, 34.39°, 35.70°, 47.56°, 56.60°, and 62.68°, which are associated with the (100), (002), (101), (102), (110), and (103) planes, respectively [[3](#_ENREF_3)]. The origin of these peaks at the specified 2θ position clearly proves the wurtzite structure of the developed ZnO nanorods, which is in agreement with PDF card no. 36-1451 obtained using Jade 6.5 software and with the previous study [[4](#_ENREF_4)]. Furthermore, as shown in Fig. 3 (b, c) and Fig. SI 5 (a, b), all peaks observed in the 3%, 5%, 7%, and 9% CO/CZO heterostructures were shifted towards a higher 2θ in comparison with those of the bare ZnO nanorods, indication a defective and relaxed lattice structure of ZnO (shown by beige areas in Fig. SI 5a). For up to 5% Co in the ZnO structure, Co substituted the Zn lattice, O lattice, and vacancy positions and revised the Zn-O and Zn-OH bonding (green area in Fig. SI 5 (b-d)). Subsequently, upon annealing, these bonds were converted into CoO phases inside the ZnO lattice structure, producing the CoO clusters on the (1010 and 101̅0) and (1120 and 11̅20) surfaces of the ZnO nanorods. Thus, the CO/CZO heterostructure was formed (blue area in Fig. SI 5 (b-d)). However, at a high percentage of Co in the heterostructure, i.e., at 9% (Fig. SI 5d), Co also started to substitute Zn_in_ and become incorporated into the interstitial sites of the lattice, which caused compressive stress, distorted the lattice (shown by beige areas in Fig. SI 5d), and further decreased the d-spacing by compressing the lattice and shifting the peaks toward higher 2θ values.


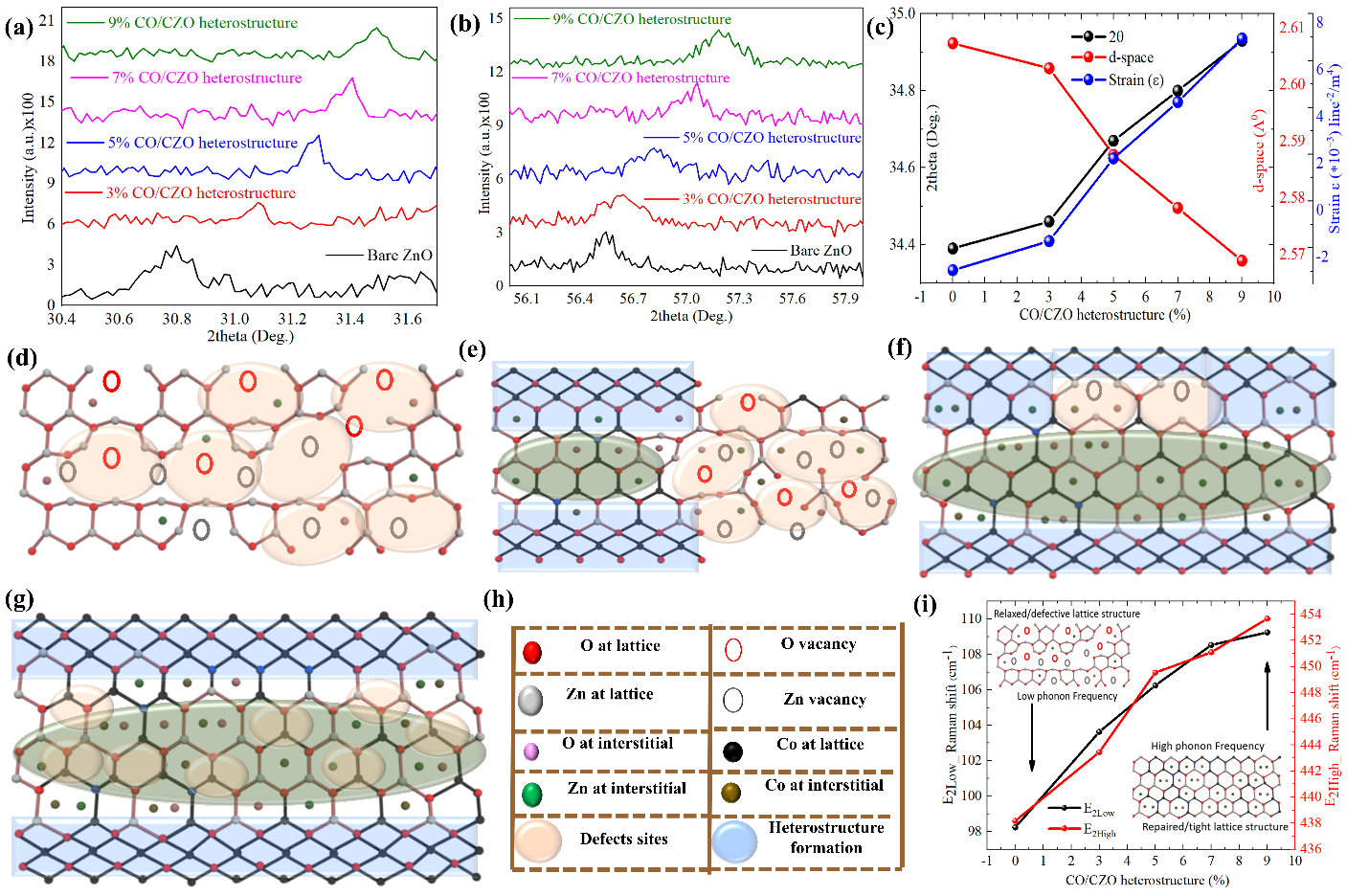


**Fig. S5.** Enlarged view of (a) (100) and (b) (110) planes observed in the XRD pattern of bare ZnO nanorods and CO/CZO heterostructure nanorods. (c) Distortion created in the lattice structure of ZnO by the compressive strain due to the Co dopant and the formation of the CoO phase, which consequently decreased the d-spacing and shifted the peaks toward higher 2θ values. Sketch depicting the lattice defects within the pristine ZnO nanorods, and the repair and distortion of the lattice structure due to CO/CZO heterostructure formation after doping with Co at various percentages: (d) defective lattice of ZnO due to the existence of O and Zn vacancies in the interstitial sites; (e) slightly repaired ZnO lattice with the 3% CO/CZO heterostructure; (f) well-repaired ZnO lattice with the 5% CO/CZO heterostructure. (g) Distortion in the lattice structure due to the interstitial substitution of Zn with Co in the heterostructure with 9% Co. Beige and blue areas show defective sites and heterostructure formation, respectively. (h) Legend for the different symbols in (d)–(g). (i) Raman spectral shift demonstrated by E_2_ (low) and E2(high) phonon modes for a relaxed and firmly bonded lattice structure of bare ZnO and CO/CZO heterostructure, respectively.

- 1. **PL spectroscopy analysis for reduction of native defects in ZnO nanorods with Co-doping**

The reduction in defect density and trapped carriers at these levels of ZnO was confirmed by PL spectroscopy analysis, where the bare ZnO nanorods exhibited a weak UV emission at approximately 384.48 nm and a strong visible defect-related emission centered at 578.12 nm, as shown in Fig. SI 5. The excited carriers were trapped by defect levels in the ZnO nanorods, resulting in the weak UV emission and strong visible deep level (DL) emission band. In the CO/CZO heterostructure, the UV emission was enhanced, while the visible DL emission was reduced in accordance with the reduction of defects. In the 5% CO/CZO heterostructure, the UV peak reached the maximum intensity, while the defect peak nearly disappeared. However, at high Co percentages, i.e., 7% and 9%, the defect peak intensity increased slightly, while the UV emission peak was slightly reduced (Fig. SI 5), confirming that 5% is a suitable addition amount of Co for the significant reduction of defect states in ZnO.


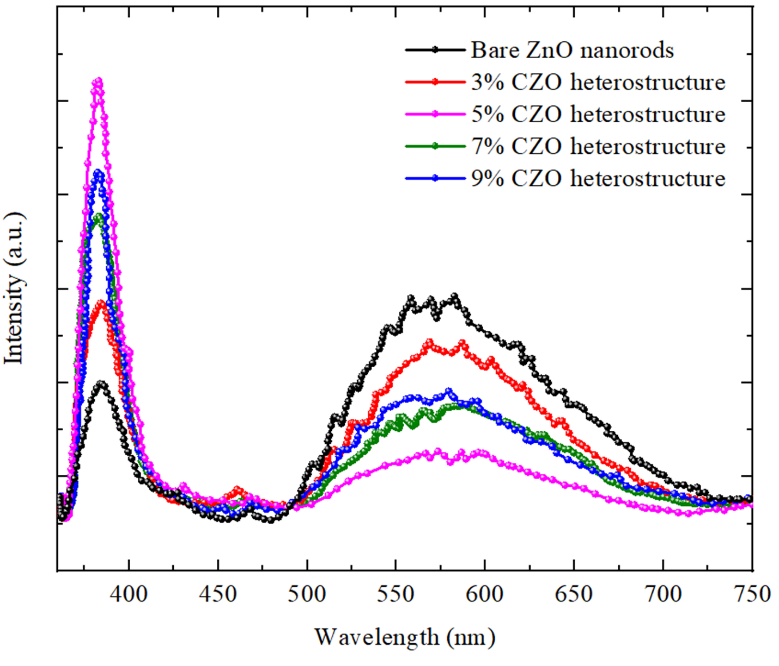


**Fig. S6.** PL spectra of bare ZnO nanorods as well as the 3%, 5%, 7%, and 9% CO/CZO heterostructures.

- 1. **Charge transport and electrochemical activities of CO/CZO heterostructure nanorods**

The electronic properties, including the flat band potential (E_FB_), carrier density (N_D_), and charge carrier transfer resistance from the electrolyte to the electrode (R_ct_), of the bare ZnO and CO/CZO heterostructure immersed in an electrolyte, can be described by Mott−Schottky (M−S) plots and EIS analysis, as shown in Fig. 4 (d-f), Fig. SI 6 (a-d) and Fig. SI 7, respectively. The M–S plot for 5% CO/CZO heterostructure (also for the 0%, 3%, 7%, and 9% CO/CZO heterostructures, as presented in Fig. SI 6 (a–d), respectively) was constructed at frequencies of 100 Hz, 316 Hz, and 1 kHz, as presented in Fig. 4d. A semiconductor/electrolyte junction forms two types of capacitance: depletion capacitance (C_dep_) and Helmholtz double-layer capacitance (C_dl_). C_dep_ is voltage-dependent, whereas C_dl_ is not. In the linear region, C_dep_ is more dominant than C_dl_; thus, the linear region was used to measure E_FB_ and N_D_. The E_FB_ value of each electrode was estimated by extrapolating the linear region of the capacitance to intersect at the potential axis.

The positive E_FB_ decreases the band-edge bending and the ɸ_B_ as determined by the potential difference between E_FB_ and the oxygen evaluation potential (E_OEP_) shown in Fig. SI 6e. In addition, the positive E_FB_ requires a lower overpotential to flatten the E_CB_ band edge (Fig. SI 6f), and it increases the charge carrier transfer efficiency by promoting the electrochemical reaction at the interface between 5% CO/CZO and the electrolyte. Next, N_D_ was estimated from the slope of the M–S plot at 1 kHz, as the M–S plot is directly related to the relative permittivity or dielectric constant (ɛ) of the semiconductor material. The ɛ vs. frequency plot of ZnO demonstrated variable ɛ values at lower frequencies and an almost constant ɛ value at high frequencies [[5](#_ENREF_5)]. Thus, the N_D_ values were calculated from the slope of the M–S plot at 1 kHz by using Eq. 1 [[6](#_ENREF_6)].

$$N_{D}=\frac{2}{\varepsilon\varepsilon_{o}e{[d\left( \frac{1}{C^{2}} \right)dV]}^{-1}}, (1)$$

where ε, ε_o_, and e, represent the dielectric constant (10 for ZnO), the permittivity of vacuum (8.85 × 10^-14^ F.cm^-1^), and electron charge (1.602 × 10^–19^ C), respectively.

In the EIS analysis, the diameter of the semicircle is related to R_ct_, where a low semicircle diameter represents a high conductivity and vice versa [[7](#_ENREF_7)]. The linear curve at lower frequencies (Fig. SI 7) represents the Warburg resistance, which describes the diffusion of ions in an electrolyte [[8](#_ENREF_8)]. The EIS Nyquist plot shows that all the CO/CZO heterostructure electrodes exhibited a smaller semicircle diameter than the ZnO electrode, indicating their lower Rct values, as shown in Fig. SI 7.

**
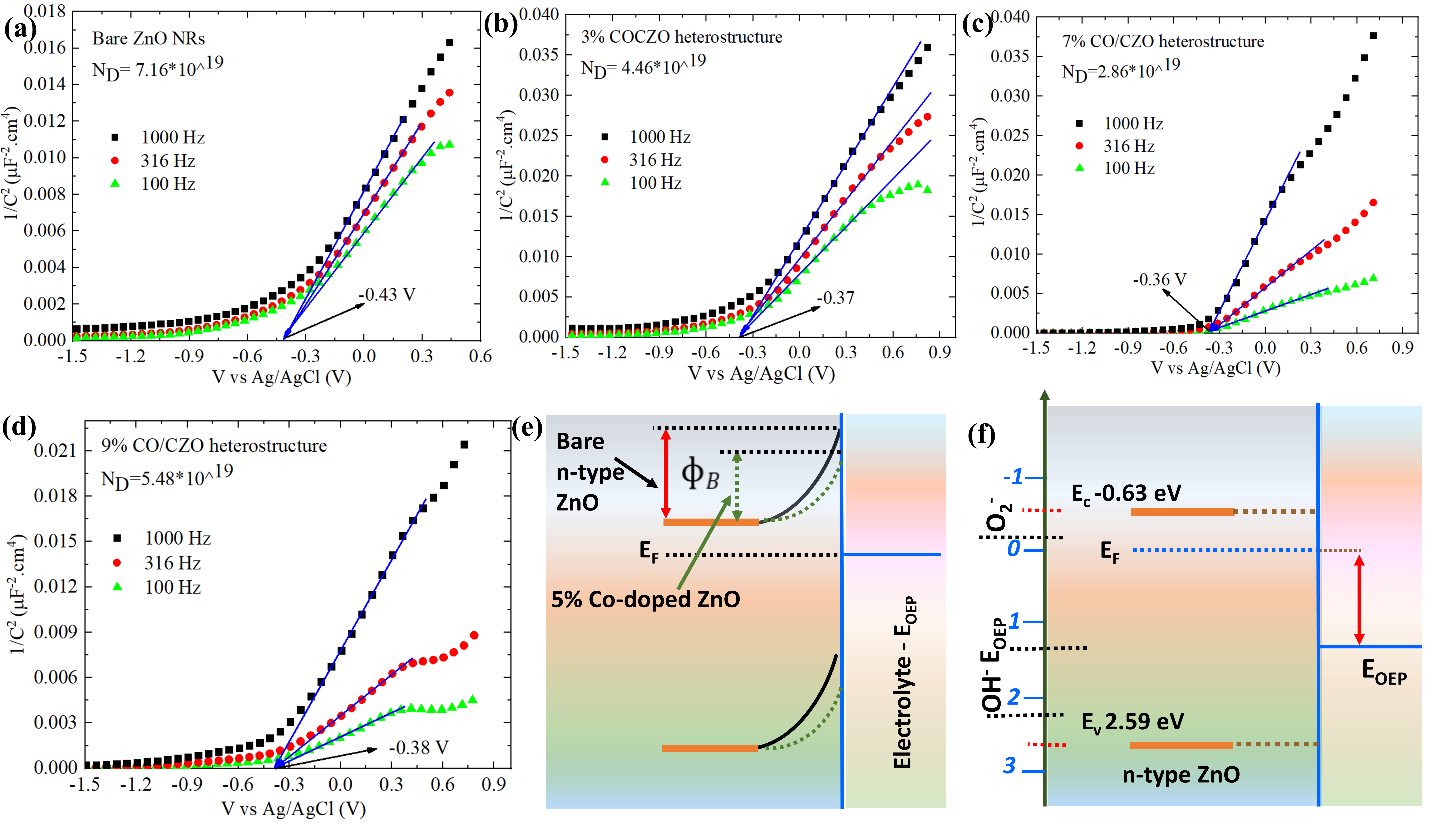
Fig. S7.** Mott–Schottky analysis of (a) bare ZnO nanorods as well as the (b) 3%, (c) 7%, and (d) 9% CO/CZO heterostructures. Energy-band diagrams of the electrode/electrolyte interface under (e) equilibrium and (f) flat-band conditions, where E_OEP_ and Φ_B_ correspond to the oxygen evolution potential and Schottky barrier height, respectively.


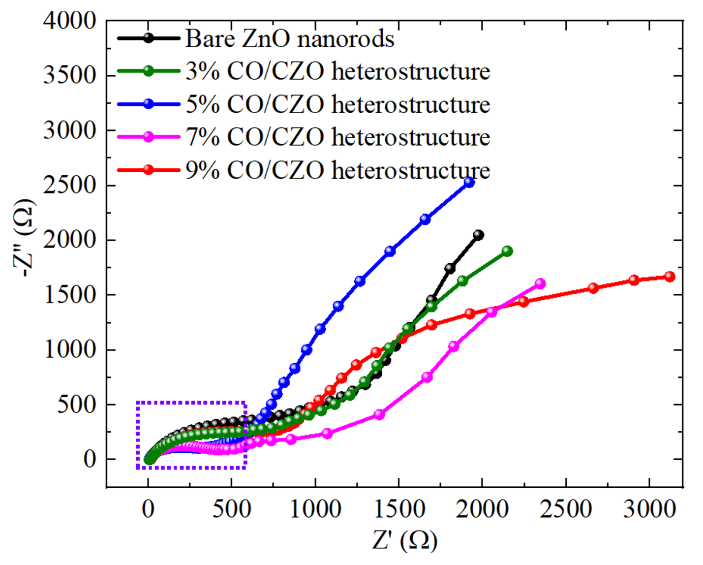


**Fig. S8.** EIS plot of bare ZnO as well as the 3%, 5%, 7%, and 9% CO/CZO heterostructures.

- 1. **Improved pH sensing performances of CO/CZO heterostructure nanorods**


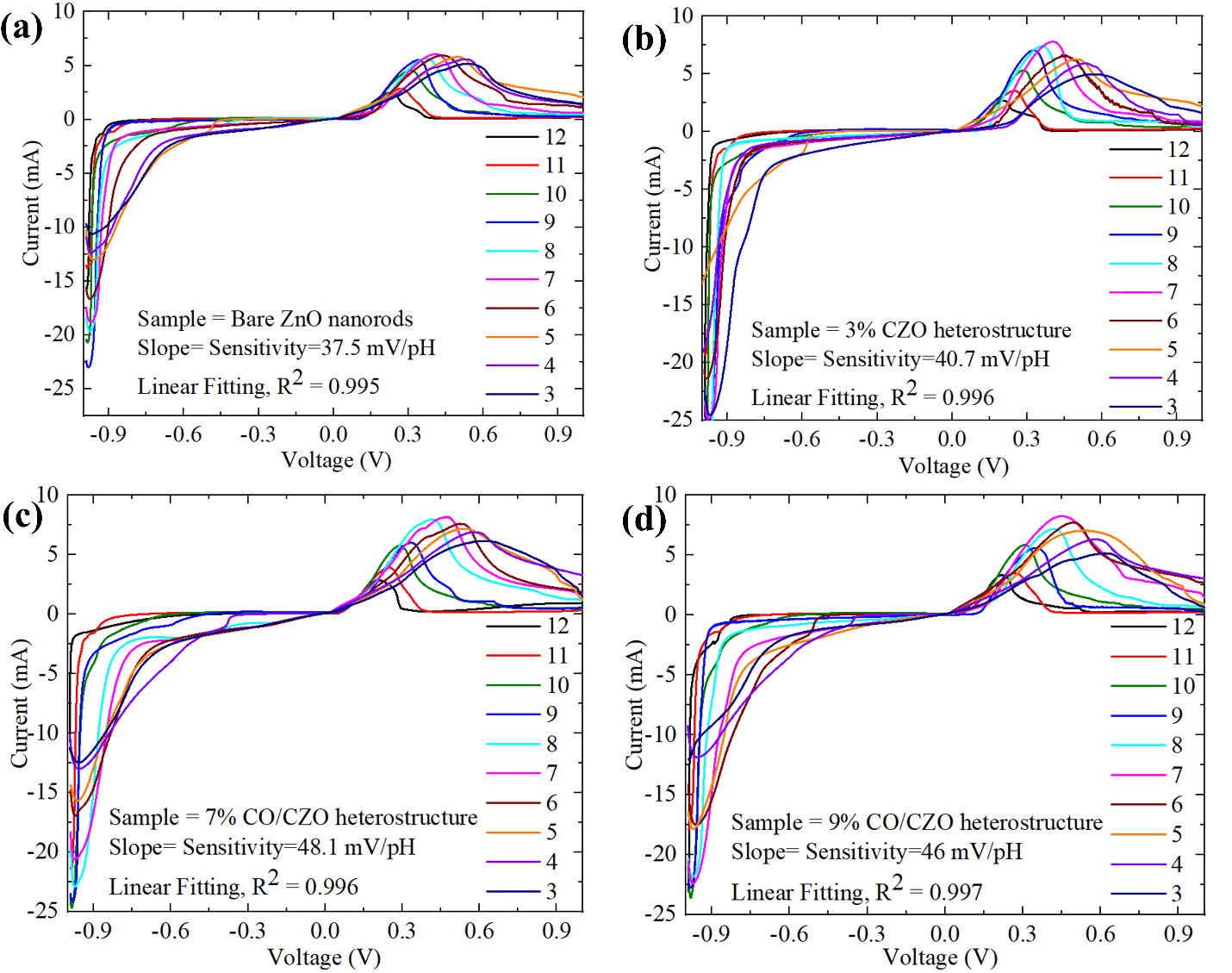


**Fig. S9.** I-V curves showing the pH sensing performances of the (a) bare ZnO nanorods as well as (b) 3%, (c) 7%, and (d) 9% CO/CZO heterostructures in various solutions of different pH values ranging from 3.0 to 12.0.


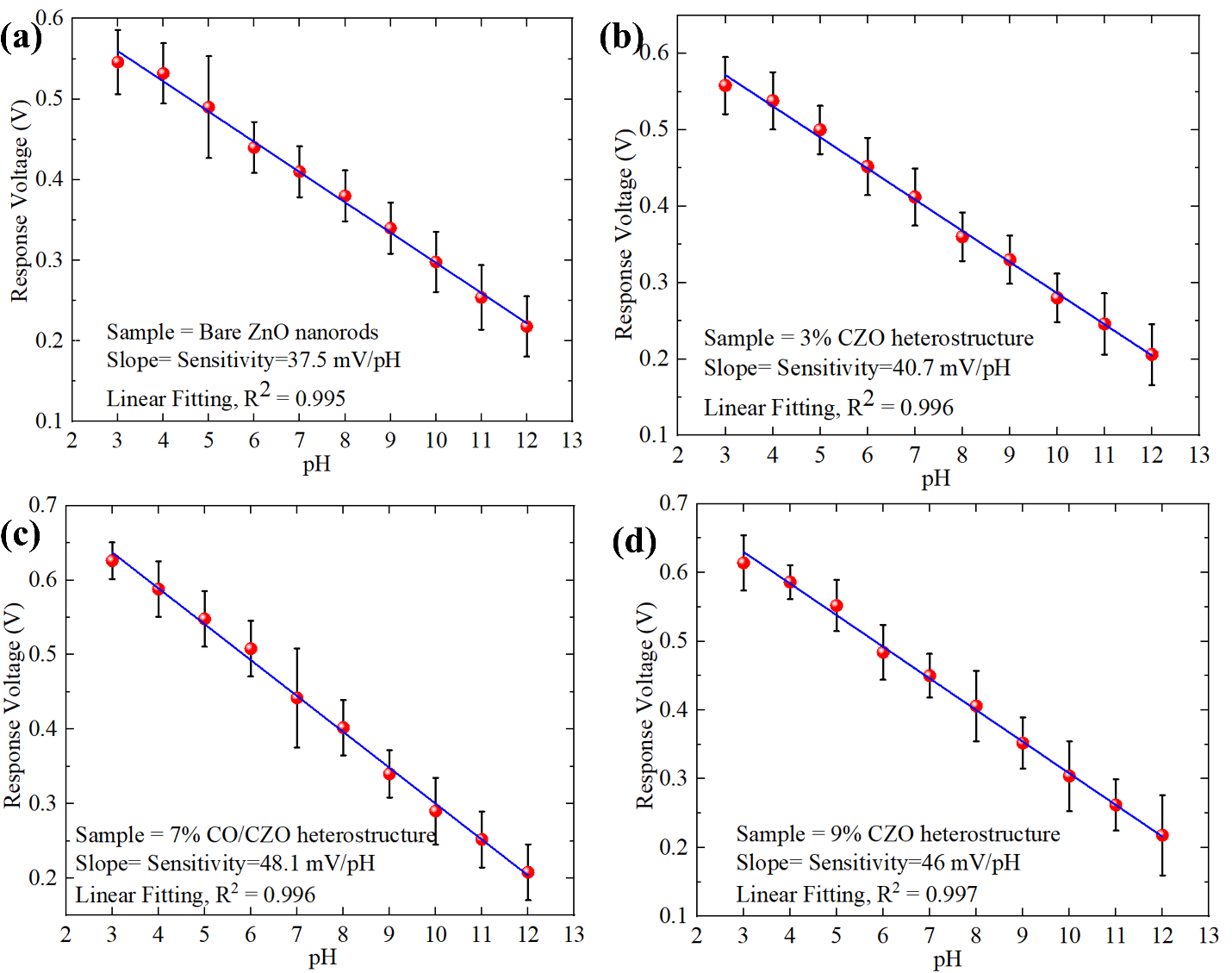


**Fig. S10.** Response voltage of the (a) bare ZnO nanorods as well as (b) 3%, (c) 7%, and (d) 9% CO/CZO heterostructures in various solutions of different pH values ranging from 3.0 to 12.0.


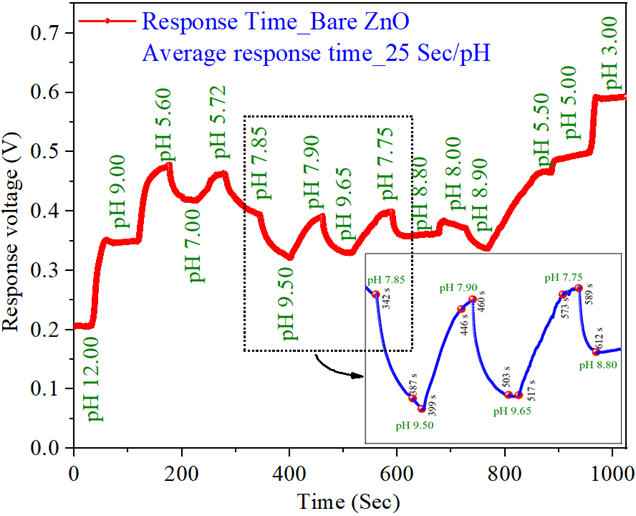


**Fig. S11.** Response time of bare ZnO based electrode.

- 1. **pH sensing performances of 5% Co-doped ZnO nanorods**

I-V measurements were performed to monitor the pH sensing performance of 5% Co-doped ZnO nanorods, as shown in Fig. SI-12a. The potential at the peak of the anodic current was used to plot the calibration curve (inset plot of Fig. SI-12a). The sensitivity of 5% Co-doped ZnO nanorods was determined to be 42.7 mV/pH. Next, its response time was determined to be 22~23 s, as shown in Fig. SI-12b. The sensitivity was determined to be 48 mV/pH from the calibration plot of the response time curve (inset plot of Fig. SI-12b). Higher sensitivity is due to using short pH range (8-12) instead of full range (3-12) solution. The obtained results demonstrated higher sensitivity and quick response time than bareZnO–based sensor, attributed to the reduction of charge density on the defects states and lower R_ct_ at electrode/electrolyte interface.


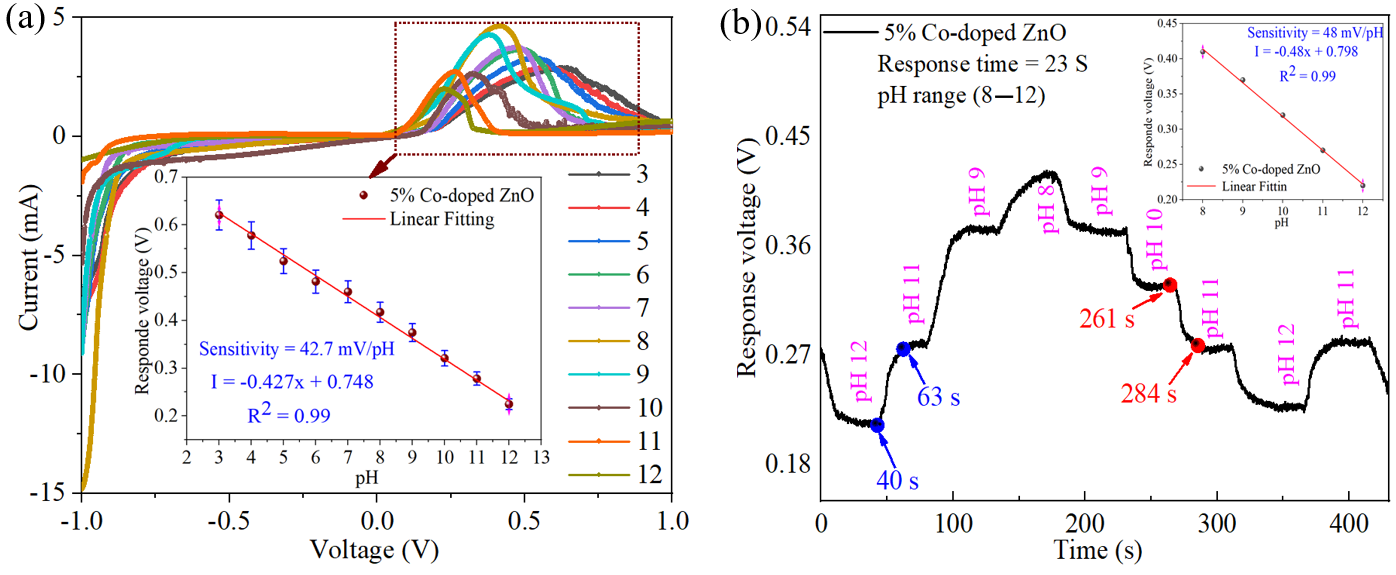


**Fig. S12** (a) I-V curves and (inset plot) corresponded response voltage showing the pH sensing performances of 5% Co-doped ZnO nanorods in various solutions of different pH values ranging from 3.0 to 12.0. (b) Response time of 5% Co-doped ZnO nanorods based electrode.

- 1. **Chemical stability of bare ZnO and the 5% CO/CZO heterostructure**


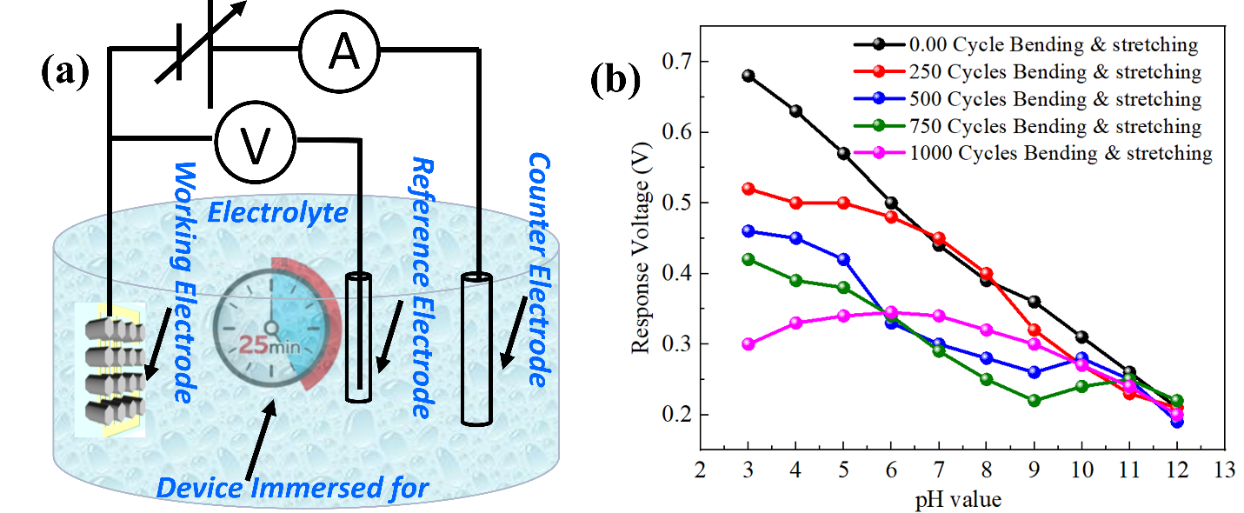


**Fig. S13.** (a)Set up used for chemical stability studies of the 5% CZO–based device and bare ZnO–based device. (b) Variation in the response voltage of the 5% CZO–based device due to the mechanical deformation.
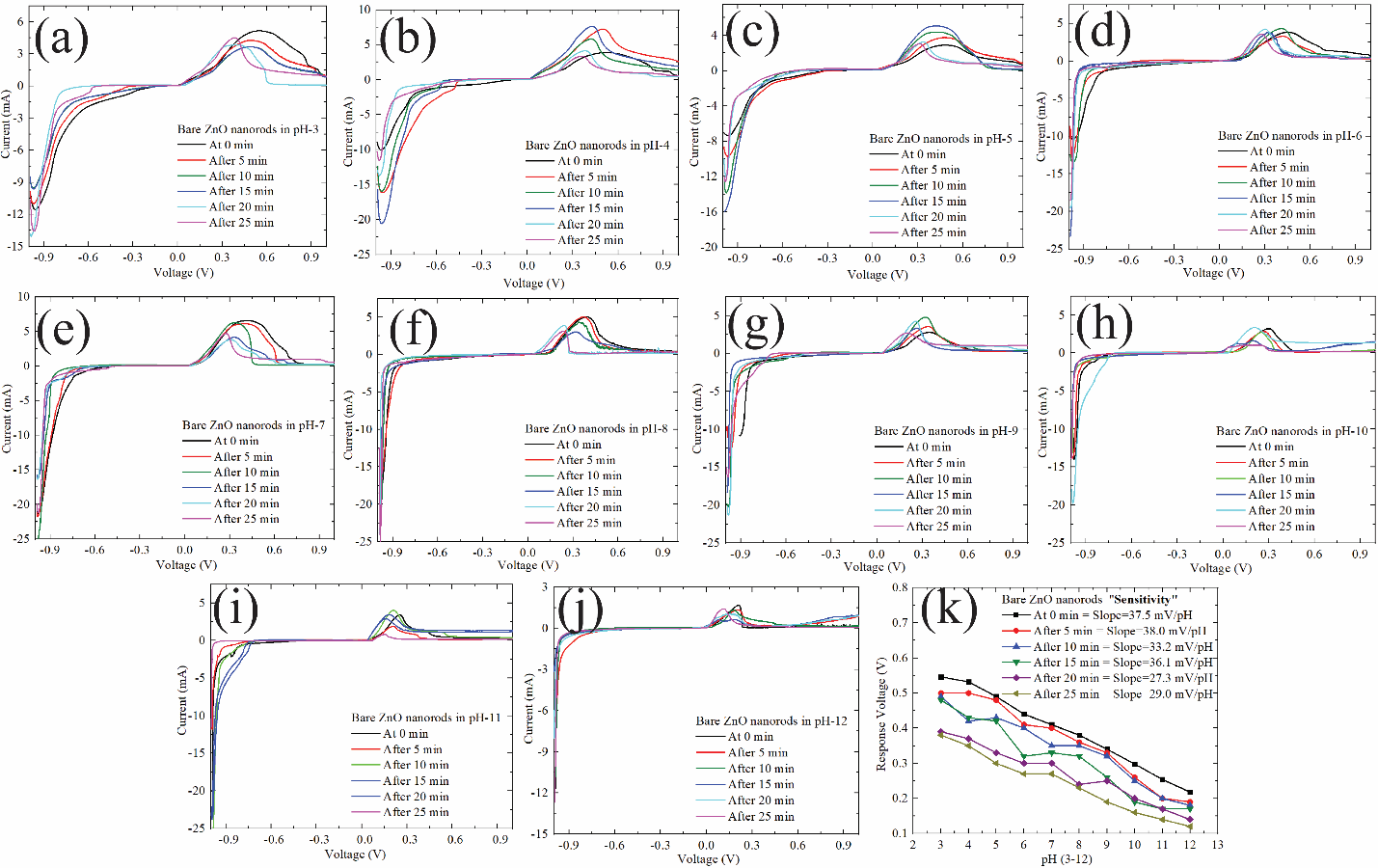


**Fig. S14.** I-V curves for chemical stability studies of bare ZnO nanorods immersed for 25 min in (a-j) various solutions of different pH values ranging from 3.0 to 12.0. (k) Variation in the response voltage of an electrode based on bare ZnO nanorods during the chemical stability test after every 5 min.


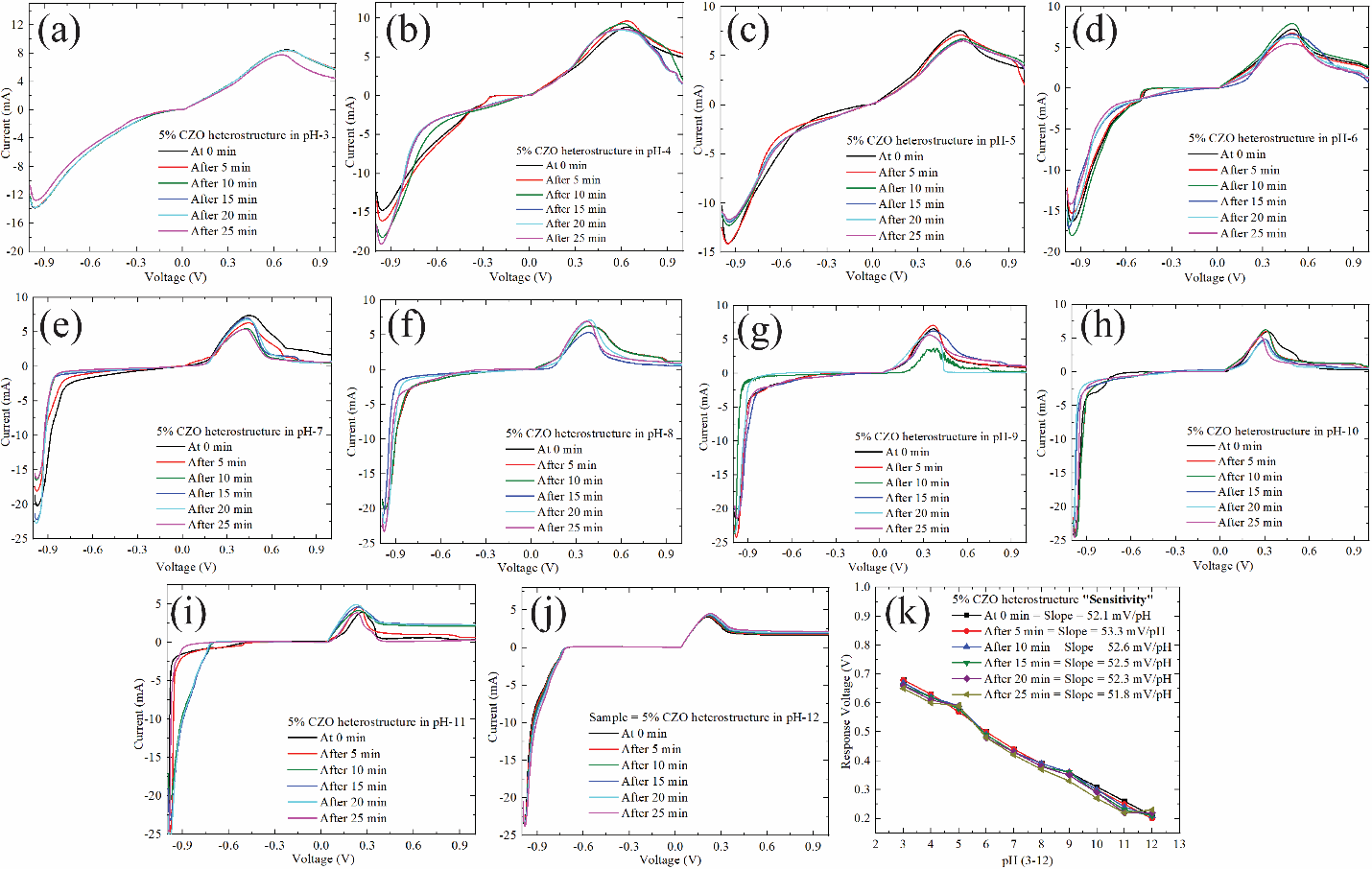


**Fig. S15.** I-V curves for chemical stability studies of the 5% CO/CZO heterostructure immersed for 25 min in (a-j) various solutions of different pH values ranging from 3.0 to 12.0. (k) Variation in the response voltage of an electrode based on the 5% CZO heterostructure during the chemical stability test after every 5 min.

- 1. **Mechanical characteristics analysis of bare PET, ZnO/PET, Co-doped ZnO/PET, and CO/CZO heterostructure/PET**

Additionally, previous studies reported that PET surfaces can crack and lose their mechanical characteristics upon exposure to an aqueous solution of different pH[[9](#_ENREF_9), [10](#_ENREF_10)]. Therefore, before performing the flexibility analysis of the 5% CO/CZO–based device, the yield stress and ultimate tensile stress of bare PET, ZnO/PET, Co-doped ZnO/PET, and CO/CZO heterostructure/PET were determined using a universal testing machine, as shown in the inset-i of Fig. 5f. ZnO addition was observed to strengthen the fracturing ability of PET to withstand high stress and strain because of the greater strength of the ZnO nanorods, which enable the reinforcement phase to produce ZnO/PET polymer composites of high strength and stiffness [[10](#_ENREF_10), [11](#_ENREF_11)]. However, bare ZnO has a deformed and relaxed structure due to several types of defects; thus, atoms of similar ionic radii are required to be doped in its lattice to repair its relaxed structure with small distortion. Therefore, Co doping enhanced the yield stress (3.34 N.m^-2^) and elongation at break (22 mm) of the ZnO/PET metal oxide–polymer composite. The corrosion resistance and fatigue life of the ZnO/PET film were further enhanced when ZnO was decorated with CoO clusters that bind together the reinforcement phases in the CO/CZO/PET composite, including the PET fiber, ZnO nanorods, Co atoms, and CoO nanoparticles, protecting the composite from chemicals, moisture, and mechanical degradation. Thus, CO/CZO demonstrated superior mechanical properties, such as yield stress (3.64 N.m^-2^), ultimate tensile stress (4.85 N.m^-2^), and elongation at break (28 mm), as well as chemical stability (1% degradation in 25 min). Hence, the CO/CZO heterostructure demonstrated excellent chemical stability (Fig. 5 (e, f) and Fig, 6 (e, f)) and functioned as a highly flexible pH and glucose electrode.


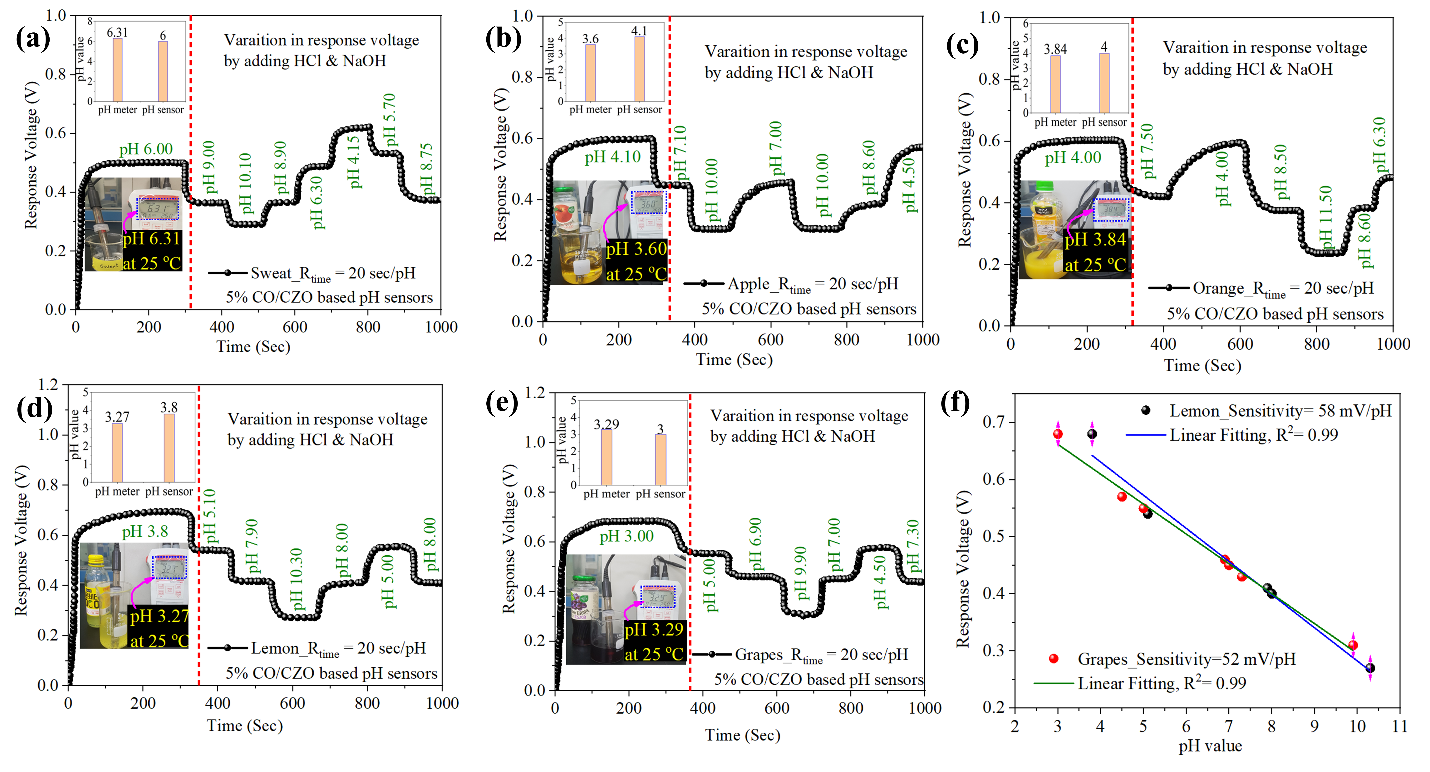


**Fig. S16.** Real-time application and monitoring of pH in (a) sweat as well as (b) apple, (c) orange, (d) lemon, and (e) grape juices. Insets: bar graphs comparing the pH results obtained using a pH meter and the developed pH sensor as well as photographs of the experimental setup. (f) Sensitivity response of the CO/CZO based device when immersed in lemon and grapes and changing their pH with HCl and NaOH.

- 1. **Reactions for glucose sensing process (SI-11-17):**

Zn^2+^ → Zn^3+^  --------------------------------(SI-11)

CoO + OH^-^ → 2 CoOOH + e^-^ ---------------------- (SI-12)

2H_2_O + e^-^ → 2OH^-^ + H_2_ ------------------------------ (SI-13)

2CoOOH + 2 OH^-^ → 2 CoO_2_ + 2H_2_O + e^-^ ----------------------- (SI-14)

Zn^3+^ + e^-^ → Zn^2+^  --------------------------------(SI-15)

C_6_H_12_O_6 (glucose)_ + 2 CoO_2_  → 2 CoOOH + C_6_H_10_O_6 (gluconolactone)_ + H_2_O_2_ ----------- (SI-16)

H_2_O_2_ → H_2_O + O_2_ + e^-^ ----------------------------- (SI-17)

- 1. **Glucose sensing performance of 5% Co-doped ZnO nanorods**


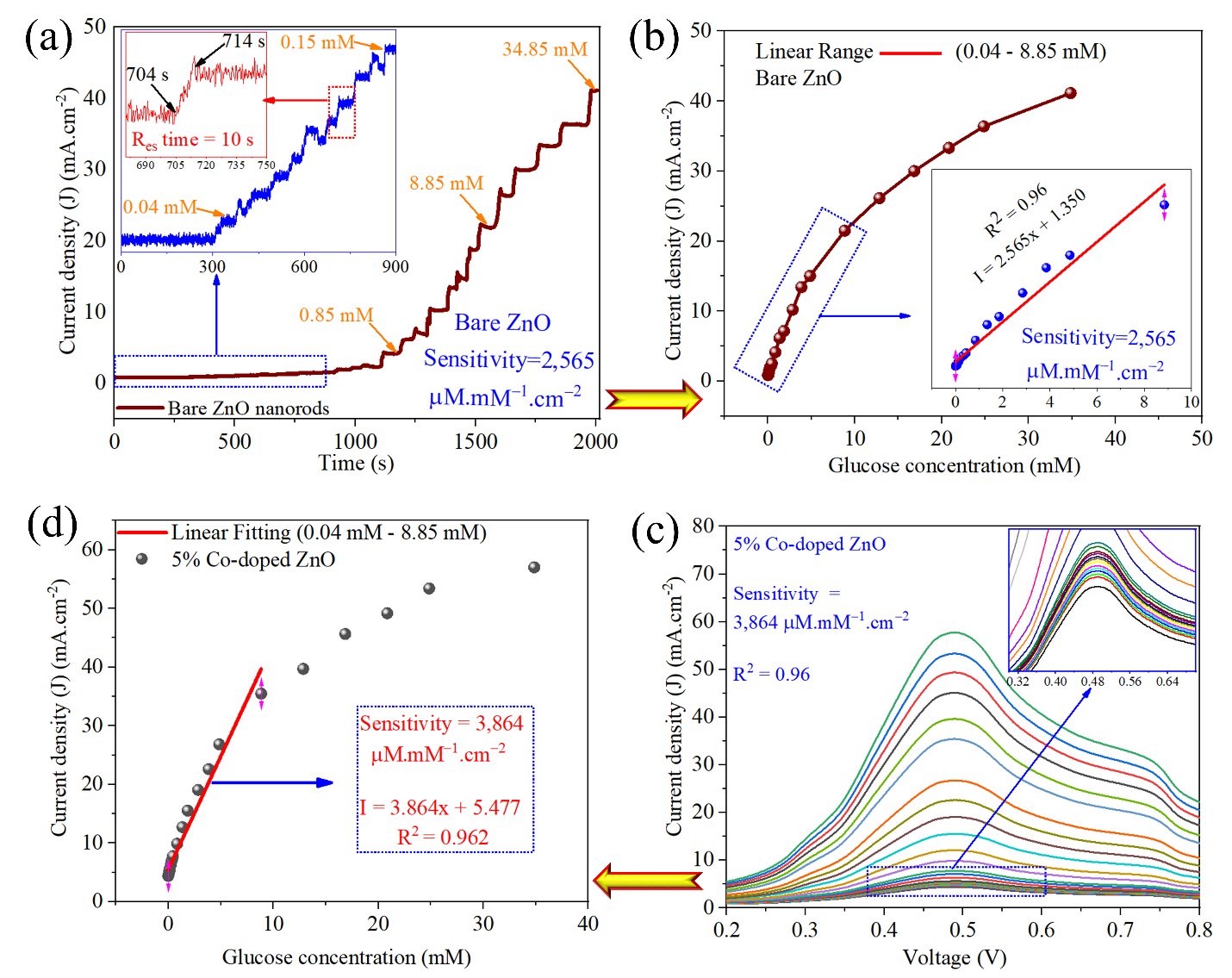


**Fig. S17.** (a) Amperometric responses of bare Zno nanorods based electrode to the glucose concentrations varies from 0.04 mM to 34.85 mM. Inset (i) is the enlarge view of the lower concentrated glucose enclosed with dash-blue rectangle and inset (ii) is the sensor response time determined to be 10 s. (b) The measured data and the calibration curve of the sensor’s amperometric response to the glucose concentration. Insets plots show the linear relationship between the oxidation peak current and concentration of glucose in the range of 0.04 mM to 8.85 mM and the corresponding regression equation. (c) I-V curve for glucose sensing performances of 5% Co-doped ZnO based electrode, where (inset) shows the enlarge of lower concentrated glucose enclosed with dash-blue rectangle. (d) The corresponded calibration plot of the linear range of 0.04 mM to 8.85 mM and regression equation.

**References:**

1. Stoltz, K.R., et al., *Optimization of the U parameter in CoO groupings in ZnO (101¯ 0) and (112¯ 0) surfaces: A DFT+ U and UPS study.* Computational Materials Science, 2021. **198**: p. 110700.

2. Zhang, W., et al., *Photocatalytic improvement of Mn-adsorbed g-C3N4.* Applied Catalysis B: Environmental, 2017. **206**: p. 271-281.

3. Kaphle, A. and P. Hari, *Variation of index of refraction in cobalt doped ZnO nanostructures.* Journal of applied physics, 2017. **122**(16): p. 165304.

4. Wang, J., et al., *Construction of 1D heterostructure NiCo@ C/ZnO nanorod with enhanced microwave absorption.* Nano-micro letters, 2021. **13**(1): p. 1-16.

5. Modwi, A., et al., *Dependence of the electrical properties of Cu-doped ZnO nanoparticles decorated by Ag atoms.* Zeitschrift für Physikalische Chemie, 2021. **235**(6): p. 745-767.

6. Karmakar, K., et al., *Stable and enhanced visible-light water electrolysis using C, N, and S surface functionalized ZnO nanorod photoanodes: engineering the absorption and electronic structure.* ACS Sustainable Chemistry & Engineering, 2016. **4**(10): p. 5693-5702.

7. Azadbakht, A., et al., *Surface-renewable AgNPs/CNT/rGO nanocomposites as bifunctional impedimetric sensors.* Nano-micro letters, 2017. **9**(1): p. 1-11.

8. Singal, S. and A.K. Srivastava, *Electrochemical impedance analysis of biofunctionalized conducting polymer-modified graphene-CNTs nanocomposite for protein detection.* Nano-micro letters, 2017. **9**(1): p. 1-9.

9. Peng, G., et al., *Effects of nano ZnO on strength and stability of unsaturated polyester composites.* Polymers for Advanced Technologies, 2008. **19**(11): p. 1629-1634.

10. Barros, A.B.d.S., et al., *The Effect of ZnO on the Failure of PET by Environmental Stress Cracking.* Materials, 2020. **13**(12): p. 2844.

11. Moskala, E.J., *A fracture mechanics approach to environmental stress cracking in poly (ethyleneterephthalate).* Polymer, 1998. **39**(3): p. 675-680.
